# Immunohistological and Ultrastructural Study of the Inflammatory Response to Perforated Polyimide Cortical Implants: Mechanisms Underlying Deterioration of Electrophysiological Recording Quality

**DOI:** 10.3389/fnins.2020.00926

**Published:** 2020-08-31

**Authors:** Shun-Ho Huang, Nava Shmoel, Maciej M. Jankowski, Hadas Erez, Aviv Sharon, Wesal Abu-Salah, Israel Nelken, Aryeh Weiss, Micha E. Spira

**Affiliations:** ^1^Department of Neurobiology, The Alexander Silberman Institute of Life Science, The Hebrew University of Jerusalem, Jerusalem, Israel; ^2^The Charles E. Smith Family and Prof. Joel Elkes Laboratory for Collaborative Research in Psychobiology, The Hebrew University of Jerusalem, Jerusalem, Israel; ^3^The Harvey M. Kruger Family Center for Nanoscience, The Hebrew University of Jerusalem, Jerusalem, Israel; ^4^Edmond and Lily Safra Center for Brain Sciences, The Hebrew University of Jerusalem, Jerusalem, Israel; ^5^Faculty of Engineering, Bar-Ilan University, Ramat Gan, Israel

**Keywords:** neural interface, foreign body response, polyimide, field potentials recordings, immunohistology, ultrastructure

## Abstract

The deterioration of field potential (FP) recording quality and yield by *in vivo* multielectrode arrays (MEA) within days to weeks of implantation severely limits progress in basic and applied brain research. The prevailing hypothesis is that implantation of MEA platforms initiate and perpetuate inflammatory processes which culminate in the formation of scar tissue (the foreign body response, FBR) around the implant. The FBR leads to progressive degradation of the recording qualities by displacing neurons away from the electrode surfaces, increasing the resistance between neurons (current source) and the sensing pads and by reducing the neurons’ excitable membrane properties and functional synaptic connectivity through the release of pro-inflammatory cytokines. Meticulous attempts to causally relate the cellular composition, cell density, and electrical properties of the FBR have failed to unequivocally correlate the deterioration of recording quality with the histological severity of the FBR. Based on confocal and electron microscope analysis of thin sections of polyimide based MEA implants along with the surrounding brain tissue at different points in time after implantation, we propose that abrupt FP amplitude attenuation occurs at the implant/brain-parenchyma junction as a result of high seal resistance insulation formed by adhering microglia to the implant surfaces. In contrast to the prevailing hypothesis, that FP decrease occurs across the encapsulating scar of the implanted MEA, this mechanism potentially explains why no correlations have been found between the dimensions and density of the FBR and the recording quality. Recognizing that the seal resistance formed by adhering-microglia to the implant constitutes a downstream element undermining extracellular FP recordings, suggests that approaches to mitigate the formation of the insulating glial could lead to improved recording quality and yield.

## Introduction

Simultaneous recordings of electrophysiological signals from many individual neurons and their stimulation in freely behaving subjects over timespans of months and years are critical to progress in basic and clinically oriented brain research (Lebedev and Nicolelis, 2017; Thompson et al., 2020). Most currently available *in vivo* multi-electrode array (MEA) implants suffer from a number of drawbacks (Jorfi et al., 2015; Seymour et al., 2017; Campbell and Wu, 2018; Wellman et al., 2018; Hong and Lieber, 2019). One major shortcoming of these devices is the deterioration of the recording yield and Field Potential (FP) amplitudes within days to weeks of implantation (Jackson and Fetz, 2007; Perge et al., 2013; Voigts et al., 2013; Lee et al., 2018). Signal quality deterioration and poor source resolution are partially alleviated by tedious spike-detecting, spike-sorting and signal averaging techniques. Aside from instances where mechanical failure leads to a decline in the yield and absolute amplitudes of the FP (Barrese et al., 2013; Prasad et al., 2014; Xie X. et al., 2014; Kozai et al., 2015b; Takmakov et al., 2015), the deterioration of the recording yield and quality is primarily attributed to inflammatory processes triggered and perpetuated by the implants that culminates histologically in chronic encapsulation of the probe by up to a 50–400 μm thick glial scar (Gray et al., 1995; Rousche and Normann, 1998; Kipke et al., 2003; Nicolelis et al., 2003; Grill and Mortimer, 2014; Wellman et al., 2019).

Three major mechanisms associated with implant-induced neuro-inflammatory processes have been suggested to account for the deterioration of the recording yield and FP amplitude over time after implantation. First, neurons are displaced from the electrode surface by a progressive increase in the thickness of the glia scar (Polikov et al., 2005; Salatino et al., 2017a). Because the amplitude of a potential generated by a point current source passively declines in the brain’s extracellular space at an approximate rate of 1/r^ x^ (where r is distance from the current source and x is in the range of 1 < x < 2 (Malaga et al., 2016; Michelson et al., 2018), the FP generated by displaced neurons decreases (Edell et al., 1992; Biran et al., 2005). Second, a self-assembled biofouling layer at the electrode surface (Sommakia et al., 2009, 2014a; Malaga et al., 2016) and the glial scar around it (Johnson et al., 2005; Polikov et al., 2005; Otto et al., 2006; Williams et al., 2007; Prasad and Sanchez, 2012) are thought to electrically insulate the electrodes from the current sources by their relative high resistivity compared to the intact brain tissue. Third, pro-inflammatory cytokines released from glia and injured neurons alter the excitable membrane properties of neurons and their synaptic connectivity in the vicinity of the electrode (Vezzani and Viviani, 2015; Salatino et al., 2017b, 2019).

Given the considerable difficulties involved in unequivocally relating recorded FP amplitudes to the spatiotemporal distributions of cellular and molecular elements of the FBR severity, the relative contribution of the different cell types comprising the inflammatory FBR and mechanisms remain elusive (Kozai et al., 2014b, 2015b; McCreery et al., 2016; Du et al., 2017; Salatino et al., 2017a; Michelson et al., 2018). Nonetheless, since the overall FBR is considered to be causally related to the deterioration of the recording over time, delaying, ameliorating or totally preventing the FBR is expected to improve the quality (amplitude, and yield), durability and stability of FP recordings. Accordingly, various approaches have been developed to acutely or chronically overcome the recording impediments imposed by the cascades of FBR pathology (Jorfi et al., 2015; Wellman et al., 2018; Hong and Lieber, 2019).

The development of cell-biological/pharmacological reagents and strategies to counteract specific stages or elements of the inflammatory cascade were marginally successful. On the one hand, the overall severity of the FBR was shown to be related to the physical properties of the implants. This includes the dimensions, flexibility and softness of the implant as well as its microarchitecture (Sommakia et al., 2014b). Smaller sizes and the use of materials with elastic moduli compatible with the elastic properties of brain tissue (Young’s moduli of 0.4–6 KPa, (Miller et al., 2000) were empirically shown to reduce the FBR level. Thus, in recent years, progress in bioengineering has led to the implementation of ultra-flexible and ultra-small platforms, with dimensions comparable to those of a single neuron (Kozai et al., 2012; Xiang et al., 2014; Fu et al., 2016; Luan et al., 2017; Zhao et al., 2017; Zhou et al., 2017; Wei et al., 2018; Guan et al., 2019; Yang et al., 2019). Standard immunohistological observations have shown that these ultra-small and flexible implants seamlessly integrate with brain tissue and that under these conditions neuronal cell bodies are found to reside in close proximity to the implant (Luan et al., 2017; Yang et al., 2019). Nevertheless, despite the fact that the impedances of these ultra-flexible platform electrodes are similar to those of conventional implants (in the range of 0.5–1 MΩ) and the seamless integration of the platforms with the brain tissue, the amplitudes of the recorded FP by the ultra-small and flexible implants were within the range of those recorded by implants that do trigger FBR.

This observation is inconsistent with the prevailing hypothesis which predicts that in the absence of a histological FBR the FP amplitudes should be larger. This inconsistency and the difficulties in establishing coherent relationships between the characteristic features of the severity of the FBR and recording qualities call for a reassessment of the leading concepts.

Here we argue that the major obstacle to a better understanding of the mechanisms underlying the attenuation of the yield and amplitude of recorded FPs over time is that traditional immunohistological studies have emphasized the overall effects of the FBR as described above, but have not focused on the micrometric relationships between implants and cells that adhere to it. Indeed, in the vast majority of studies designed to explore the electrode/tissue structural interface, the implants had to be extracted from the brain tissue prior to thin sectioning for histological examination. This unavoidably defaced this micrometric junction making it impossible to examine the structural relationships between the implant and the tissue around it. In a relatively small number of studies, including the new generation of ultra-flexible electrodes, immunohistological analysis has been conducted along with the electrode. Unfortunately these studies use low resolution imaging and cannot resolve the required level of detail. While using scanning electron micrographs (SEM) of extracted implants, a number of studies have documented the presence of cells adhering to the platform surfaces (Holecko et al., 2005; Barrese et al., 2016; Du et al., 2017), nevertheless, examination of the surfaces of extracted implants cannot establish the extent and nature of the adhering cells.

The present study was designed to improve understanding of the spatiotemporal relationships between cortical implants and induced inflammatory cell cascades by centering on the micrometric relationships between the adhering cells and the implant surface. To that end we fabricated a relatively large footprint perforated polyimide-based (PI) multielectrode array (MEA) platforms (Perforated Polyimide based Multielectrode array Platform-PPMP) that can be thin sectioned along with the surrounding tissue. Using immunohistological and transmission electron microscopy (TEM) images we analyzed in time and space the interface formed between the implant and the surrounding parenchyma. The structural observations were then complemented by electrophysiological recordings.

Based on these observations we suggest that FPs generated by neurons are abruptly attenuated or blocked locally at the interface with the implant electrodes by microglia that adhere to the implant and form a high seal resistance barrier. We posit that this barrier is the major underlying mechanism governing the deterioration and blockade of FP recordings by implants.

## Materials and Methods

### Electrode Design and Fabrication

Perforated Polyimide based MEA Platforms (PPMPs) were constructed using standard photolithography fabrication methods as follows. First, an aluminum releasing layer was sputtered on a 3-inch silicon wafer (University Wafer, United States), followed by a spin-coated 15 μm thick polyimide layer (PI 2610, HD Microsystems, Germany) that served as an insulating layer and the main mechanical backbone of the platform. A triple metal layer of Cr/Au/Cr (20/120/20 nm) was then patterned and e-beam evaporated as interconnects, pads and scribe-lines. Next, a second insulating layer of polyimide (1 μm) was spin-coated, followed by the deposition of a 1 μm SiO_2_ with Plasma Enhanced Chemical Vapor Deposition (PECVD). A photoresist layer (1.5 μm) was patterned to define 25 μm molds for electroplating the electrodes and pads, dry etched by RIE through the SiO_2_ and the one micrometer PI layers. After removal of the top Cr layer by wet etch, planar electrodes with diameters of 25 μm and a thickness of ∼2.5 μm were electrodeposited. An additional 300 nm of SiO_2_ was deposited with PECVD and a photoresist layer was used to define the pattern of the platform with the perforation feature. The platforms were then released from the wafer by anodic metal dissolution and thoroughly rinsed in distilled deionized water.

After release, sixteen channel Omnetics connectors (A79042-001) were glued with conductive material (124-08 LVC, Creative Materials, United States) to the pads. To mechanically stabilize the structure, the junction between the PI platform and the connector was further encapsulated with biocompatible medical grade epoxy (353 ND, Epoxy technology).

**FIGURE 1:**
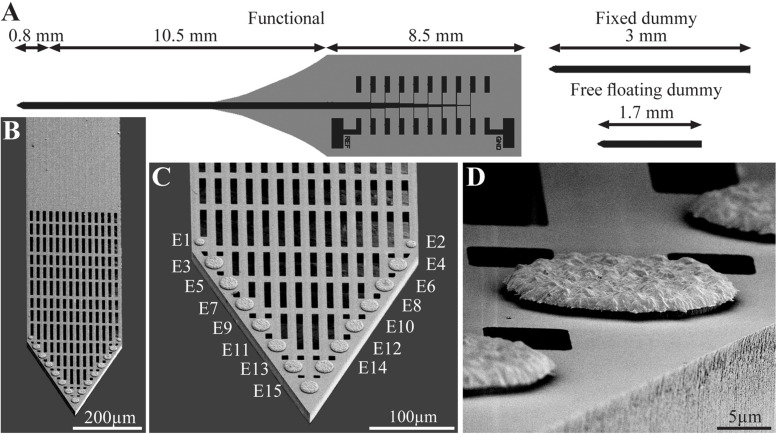
The perforated polyimide based MEA platform (PPMP). **(A)** Schematic drawing of the functional PPMP (left hand side), fixed and free floating dummy implants (right hand side). The functional PPMPs were constructed of single 280 μm wide and 16 μm thick shanks. The shanks were subdivided into a 0.8 mm long distal perforated part and a 10.5 mm solid proximal part. Gold interconnects, insulated by a PI layer, terminated by output pads at the proximal end of the shaft. **(B)** Scanning electron microscope image of the perforated and solid parts of a PPMP shank. **(C)** Enlargement of the platform tip carrying 15 planar electrodes. **(D)** Enlargement of a planar gold electrode.

Using the above process flow, two types of single shaft, 280 μm wide and 16 μm thick platforms were fabricated: (a) functional platforms for electrophysiological recordings. These platforms carried 15 planar electrodes that were terminated with output pads at the proximal end of the shaft (Figure 1); (b), “dummy” platforms for immuno- and electron-microscopic studies. These differed from the functional platforms solely in that the output pads were not fabricated. To optimize the position of the gold electrodes they were deposited along the edges of the tapering tip of the platform (Lee et al., 2018) with a center to center distance of 45 μm. The tip of both the functional and the dummy MEA platforms were divided into two segments: a 0.8 mm long perforated distal part and a 10.5 mm long proximal solid shaft (Figure 1). The perforated segment tapered to form a sharp tip. The width of all the rectangular perforations (for short perforations) was 7–8 μm and the lengths of the different perforations were 65, 47 and 44 μm (Figure 1). The perforated configuration of the platform reduced the projected solid surface area of the perforated part by 35% and allowed extension or migration of branches and cells throughout the platform. The perforations permitted diffusion of ions and molecular components across the platform. In addition, each pore approximately doubled the PI surface to which cells could adhere.

To mechanically stabilize the PI platform for implantation, the output pad area and a 6.8 mm long segment of the PI shank were glued to a 300 μm thick kapton tape leaving the most distal 1.6–2 mm PI shank free for insertion (Supplementary Figure S1).

### Animals and PPMP Implantation

All procedures in the present study were approved by the Committee for Animal Experimentation at the Institute of Life Sciences of the Hebrew University of Jerusalem. All procedures were carried out in accordance with the approved guidelines.

### Implantation of Functional Electrodes

**FIGURE 2:**
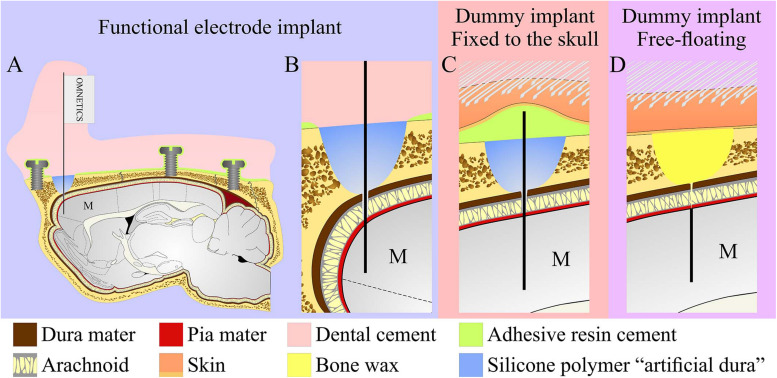
Schematic drawing of the three modes of perforated polyimide MEA platform (PPMP) implantations. **(A,B)** View of a functional PPMP implant in the sagittal plane. A PPMP attached to an omnetics connector inserted into the motor cortex (M). The dura is protected by silicone polymer (“artificial dura”). The implant is anchored to the skull with 6 screws and adhesive resin dental cement. The holding body of this transcutaneous implant was made of acrylic dental cement. **(C)** Fixed dummy PPMP implant in the sagittal plane. The dura is protected by a silicone polymer and the electrode platform is fixed to the skull with adhesive resin cement. The skin above the implant is closed after implantation, creating a subcutaneous implant configuration. **(D)** Free-floating dummy implant in the sagittal plane. The short implant is inserted into the motor cortex. The dura and opening in the bone are protected by bone wax and the skin above the implant is closed after implantation creating a subcutaneous implant configuration.

The implantation of the functional PPMP (Figure 2) for chronic wireless electrophysiological recordings in freely moving rats was divided into two stages: (1) preparation of a base for the platform implant, and 14–28 days later (2), implantation of the recording platform into the brain tissue. The separation between the invasive parts of the surgery (1), from the very delicate part of platform implantation (2), allowed us to start the wireless electrophysiological recordings soon after the PPMP implantation in naturally behaving animals in which the major tissue damages inflicted in stage (1) were completely healed.

**Stage (1):** Female Sprague Dawley rats (240–340 g) were initially anesthetized in an induction chamber with Sevoflurane (8% in air, Piramal Critical Care Inc., Bethlehem, PA, United States) using a SomnoSuite^®^ Low-Flow Anesthesia System (Kent Scientific Corporation, Torrington, CT, United States). The head was shaved and the rat was placed in a stereotaxic instrument with a mask for gas anesthesia (David Kopf Instruments, CA, United States). The Sevoflurane concentration was slowly adjusted to a level of about 3.7% and maintained at this level throughout surgery. The surgical level of anesthesia was verified by a lack of pedal-withdrawal reflex and breathing rate. Body temperature was controlled with a closed loop heating system with a rectal probe. The eyes were protected with a thick layer of Vaseline and the skin on the head was disinfected with povidone-iodine solution (10%, equivalent of 1% iodine, Rekah Pharm. Ind. Ltd., Holon, Israel). To prevent postoperative pain, the rats received a subcutaneous injection of Carprofen 50 mg/ml (5% W/V, Norocarp, Norbrook, Newry, Northern Ireland, United Kingdom) using a dose of about 12 mg/kg during surgery.

A 1.5–2 cm longitudinal cut of the skin on the head was made and the dorsal surface of the skull was exposed. The connective tissue covering the bones was removed and the bones were cleaned with sterile saline. Then the surface of the bones was treated with a 15% hydrogen peroxide solution (Sigma Aldrich Inc., St. Louis, MO, United States) and the area was flushed with sterile saline after 10–20 s. When the surface of the skull was clean and dry, a reference point for the entrance of the recording electrodes was marked to target the motor cortex at the following coordinates: AP: + 3.5 to + 5.0 mm; ML: + 2.5 mm from the Bregma. Subsequently, 6 openings for supporting screws were drilled and screws were mounted in the skull. One screw soldered to a ground wire was placed in the left frontal bone. The screws were fixed together and to the bone first with resin and then with acrylic dental cement (Super-bond C&B, Sun Medical, Moriyama, Shiga, Japan; Coral-Fix, Tel Aviv, Israel) to form the base of the implant. The ground wire was connected to a small female connector (853 Interconnect Socket, MILL-MAX MFG. CORP., New York, United States) embedded in the cement in the front of the implant base to create an easy, low impedance connection to the ground wire during recordings. A thin polyimide tube was placed on the skull vertically above the projected electrode implantation site and cemented together with the rest of the implant base.

The wounds were cleaned and treated *in situ* with antibiotic ointment (synthomycine, chloramphenicol 5%, Rekah Pharm. Ind. Ltd., Holon, Israel) and Bismuth subgallate (Dermatol, Floris, Kiryat Bialik, Israel). The skin was sutured (Nylon, Assut sutures, Corgémont, Switzerland) in the anterior part of the implant with one or two sutures to stretch the skin around the base of the implant. Rats received an intraperitoneal injection of Enrofloxacin antibiotic 50 mg/ml (5% W/V) at a dose of 15 mg/kg diluted with saline for a total volume of 1 ml (Baytril, Bayer Animal Health GmbH, Leverkusen, Germany). For the first two days after surgery, Meloxicam (Loxicom 1.5 mg/ml, Norbrook, Newry, Northern Ireland, United Kingdom) was dissolved in palatable wet food and served to the rats in their home cages (0.6 mg in one portion given every 24 h). Rats were allowed at least 2–4 weeks of recovery post-surgery before starting the next procedure. After surgery, the animals were housed individually to prevent damage to the implants.

**Stage (2):** When the wounds had completely healed, 14–28 days after stage (1), the rats were subjected to the implantation of recording platform.

As described above, the rats were initially anesthetized in an induction chamber with Sevoflurane (8% in air) and transferred to a stereotaxic instrument with a mask for gas anesthesia. The Sevoflurane concentration was adjusted to a level of 3.7% and maintained at this level throughout the procedure. The eyes were protected with Vaseline and body temperature was controlled using a closed loop heating system with a rectal probe.

The dental cement above the implantation site marked by the polyimide tube was removed using a dental drill until the skull was exposed. The craniotomy was performed by drilling, and the dura was gently resected (0.3–0.5 mm long incision). Electrodes were inserted into the brain tissue using a micromanipulator (to a depth of 1.1–1.5 mm below the brain surface) at a rate of 100 μm/min. Neural activity was monitored during insertion to identify the optimal depth. The craniotomy was sealed with elastic silicone polymer (Duragel, Cambridge Neurotech, United Kingdom) and the electrodes were fixed to the base of the implant with acrylic dental cement.

To prevent postoperative pain, the rats received a subcutaneous injection of Carprofen 50 mg/ml (5% W/V) at a dose of about 12 mg/kg. For the first two days after surgery, Meloxicam dissolved in palatable wet food was served to the rats in their home cages (0.6 mg in one portion given every 24 h).

### Implantation of PPMP Platforms for Immunohistological and Electron Microscope Studies

For economic (the cost of fabricating and packaging functional PPMPs) and technical reasons related to the cryosectioning of the skull and the screws (Figure 2), we mainly used “dummy” platforms for the immunohistological and ultrastructural studies. Structurally the dummy platforms only differed from the functional platforms in that the proximal part of the output pads was not fabricated (Figure 1). Two modes of dummy platform implantations were used: (a) implantation of dummy platforms into the cortex without fixing (gluing) the electrode to the skull, dubbed “free-floating dummy platforms” (Figures 1, 2) and (b), implantation of dummy platforms that were fixed to the skull (Figures 1, 2). The latter configuration better imitates the configuration of the functional platform implants in that the shank is exposed to the micromotion of the brain tissue.

### Free-Floating Dummy Electrode Implantation

A 1–1.5 cm longitudinal cut of the skin on the head was made and the anterior, dorsal surfaces of the skull were exposed. Two craniotomies, one in the left and the other in the right frontal bones, were performed at the desired reference points and the dura was gently resected (0.3–0.5 mm long incision). The 1.7 mm long platforms (only the shank without the pad output, Figure 2) were slowly inserted into the motor cortex. The electrodes were released from the holder and the craniotomy was sealed with melted bone wax (W810, Ethicon, Belgium). The wound was treated *in situ* with antibiotic ointment (Synthomycine, chloramphenicol 5%) and sutured with nylon sutures. The rats received an intraperitoneal injection of Enrofloxacin 50 mg/ml (5% W/V) and meloxicam in palatable wet food as described above.

### Fixed-to-the-Skull Dummy Platform

Similar to the free floating platform, a 1–1.5 cm longitudinal cut of the skin on the head was made and anterior, dorsal surfaces of the skull were exposed. The connective tissue was removed from the bones and the surface of frontal bones was cleaned and dried. Reference points for the entrance of the dummy platforms were marked, and the bones covered with resin dental cement (Super-bond C&B, Sun Medical, Moriyama, Shiga, Japan). Two craniotomies at the desired reference points were performed and the dura was gently resected (0.3–0.5 mm long incision) and the platforms were slowly inserted into the brain tissue using a micromanipulator. The craniotomy was sealed with elastic silicone polymer (Duragel, Cambridge Neurotech, United Kingdom). In contrast to the free floating dummy implants, this implant was fixed to the prepared base with the same resin dental cement (Figure 2). The dummy platform was released from the holder and cut just above the cement. All sharp edges created after cutting off the electrode shaft were covered with dental cement to form a smooth dorsal surface on the implant. The wound was treated *in situ* with antibiotic ointment (Synthomycine, chloramphenicol 5%) and sutured with nylon sutures. The rats received an intraperitoneal injection of Enrofloxacin 50 mg/ml (5% W/V) at a dose of 15 mg/kg diluted with saline to 1 ml. For the first two days after surgery, Meloxicam in palatable wet food was served to the rats in their home cages (0.6 mg in one portion given every 24 h).

### Electrophysiology

Voltage recordings from freely moving rats were amplified and digitized using a 32-channel multichannel system wireless amplifier (W2100-HS32 Multichannel systems, a division of Harvard Bioscience, Inc.) connected to the PPMP by fifteen channel Omnetics connectors (A79042-001). As a ground reference we used the screws attaching the MEA platform to the skull (Precision Technology Supplies Ltd. United Kingdom). The sampling rate was 20 kHz and a 5 Hz high-pass and a 3000 Hz low-pass filters were applied for local field potential (LFP) and single-unit recordings. Spike sorting was performed using the fully automatic spike-sorting implementation described in Chaure et al. (2018). Electrode impedances were measured *in vitro* before implantation and *in vivo* at 1 kHz using the nanoZ impedance tester (Plexon).

### Tissue Processing for Immunohistology and Electron Microscopy

Rats implanted with “fixed to the skull” or “floating” dummy MEA platforms were sacrificed at 1 hour, 1 day, 3 days, 1, 2, 4 or 8 weeks after implantation. Rats implanted with functional platforms were sacrificed when the Omnetics connectors linking the electrodes and the telemetric amplifier failed to provide stable electrophysiological recordings. For brain tissue fixation, individual rats were deeply anesthetized with isoflurane (Piramal, United states) followed by an IP overdose injection of Pentel (4.5 ml per 250 g rat, CTS Group, Israel). When they stopped breathing, the rats were transcardially perfused by phosphate buffer saline (PBS). This was followed by a 4% paraformaldehyde in PBS (PFA, Sigma-Aldrich) perfusion at a rate of 10 ml/min for 40 min. for immunohistological processing or by 2.5% glutaraldehyde (Agar scientific) and 2% paraformaldehyde perfusions for transmission electron microscope processing.

Next, the skulls of rats implanted with untethered dummy electrodes and destined for immunohistological examination or TEM analysis were removed and the implanted brain was post-fixed at 4°C for an additional 12–24 h. either in PFA (for immunohistology) or in glutaraldehyde/paraformaldehyde for TEM examination. Thereafter, the PFA fixed and exposed brains destined for immunohistological examinations were washed in PBS and incubated for 1–3 days in a 30% sucrose solution in PBS at 4°C.

To enable the handling of rat brains implanted by tethered MEA platforms (dummy or functional electrodes) for immunohistological observations without moving the electrode relative to the brain tissue, during skull removal, we softened the skull by incubation in a decalcification solution (0.25 M EDTA (J.T. Baker 8993-1) and 0.0325 M NaCl, PH 7.4) for 5–7 days. Next, the brain tissue was exposed to a 30% sucrose solution by removal of the frontal (the area around the olfactory bulbs) and caudal part (cerebellum) of the skull with a dental drilling disc.

### Cryosectioning and Immunohistology Labeling

To prepare for cryosectioning of the brain tissue, a cubic shaped portion of tissue, (approximately 1 × 1 × 1 cm) with the Perforated PI platform at its center was isolated. For the tethered platforms, the softened skull remained attached to the brain tissue. The isolated brain piece (together with the attached skull) was placed in a freezing medium (Tissue- Plus O.C.T. Compound, Scigen) and frozen at −80°C. The frozen tissue along with the implanted platform was then horizontally sectioned into 40 μm thick slices using a Leica CM1850 Cryostat. Individual slices were collected and placed in 24 well plates containing PBS.

The tissue slices were then incubated in blocking solution (1×PBS, 1% heat-inactivated horse serum (Biological Industries), 0.1% Triton X-100 (Sigma Aldrich)) for 1 hour at room temperature (RT) under gentle shaking. Next, the slices were incubated with a diluted primary antibody for 3 hours at room temperature (RT) and washed 3 times with the blocking solution. This was followed by a 1-hour incubation at RT with the diluted secondary antibody after which the slices were washed with the blocking solution 3 times and stained with the nuclear marker DAPI (Sigma–Aldrich, 1 mg/ml 1:1000) for 15 min at RT. After washing with the blocking solution and PBS the slices were mounted on Superfrost Plus Slides (Thermo Fisher Scientific) and sealed by a Vectashield (VE-H-1000 -Vector Labs) mounting medium. Meticulous examination of the prepared tissue slices by confocal microscope optical sections revealed that the antibodies penetrated the tissue to homogeneously stain the target cells.

Neurons were concomitantly labeled with two antibodies: one for neurite labeling (mouse anti-Neurofilament 160/200 monoclonal antibody (Sigma Aldrich N2912, 1:10000-1:20000) and the other for neuronal nuclei(mouse anti-NeuN monoclonal antibody (Merck MAB377, 1:200)). Astrocytes were labeled with chicken anti-glial fibrillary acidic protein (GFAP) polyclonal antibodies (Thermo Fisher PA1-10004, 1:1000). Microglia were labeled using rabbit anti-Ibl-1 monoclonal antibody (Abcam ab178846, 1:2000). For the secondary antibodies we used goat anti- mouse Alexa 488, goat anti- chicken Alexa 647 (Thermo Fisher A-11001 and A21449 respectively, 1:100) and sheep anti-rabbit Cy3 (Sigma–Aldrich C2306, 1:100). To confirm that the Iba-1 antibody labeled resident microglia and did not infiltrate the macrophages we co-labeled the Iba-1 positive cells (using goat anti Iba-1 antibody (Abcam ab5076, 1:125) with rabbit polyclonal antibody for rat TMEM119 that recognizes microglia specific transmembrane proteins (Synaptic Systems GmbH, 1:100, 400 203). To co-label microglia and dividing cells we used goat anti Iba-1 (Abcam ab5076, 1:125) together with rabbit anti polyclonal KI67 (Abcam, ab15580, 1:200). For the secondary antibodies, we used donkey anti goat Alexa405 (Abcam ab175665, 1:100) and sheep anti-rabbit Cy3 (Sigma–Aldrich C2306, 1:100).

### Microscopy

Confocal image stacks of the immunolabeled slices were acquired with an Olympus FLUOVIEW FV3000 confocal scan head coupled to an IX83 inverted microscope, using a 20× air objective (NA = 0.75). Scanning was done in sequential mode in 2 phases. In one phase, DAPI was excited using the 405 nm laser and its emission was acquired in the 415 to 470 nm range using a spectral detector, whereas Cy3 was excited using the 561 nm laser and its emission was acquired in the 570–630 nm range using a second spectral detector. In the second phase, Alexa488 was excited with the 488 nm laser and its emission collected in the 500 to 540 nm range, while the Alexa647 was excited using the 640 nm laser, and its emission was collected in the 645 to 745 nm range, again using the two spectral detectors. A non-confocal transmitted light image was also acquired, and served to visualize the electrode.

For the co-staining of Iba1 and TMEM-119 or Iba-1 and KI67 we used sequential mode: Alexa405 was excited using the 405 nm laser and its emission was acquired in the 420 to 495nm range using a spectral detector, whereas Cy3 was excited using the 561 nm laser and its emission was acquired in the 575–650nm range using a second spectral detector. A non-confocal transmitted light image was also acquired, and served to visualize the electrode.

**FIGURE 3:**
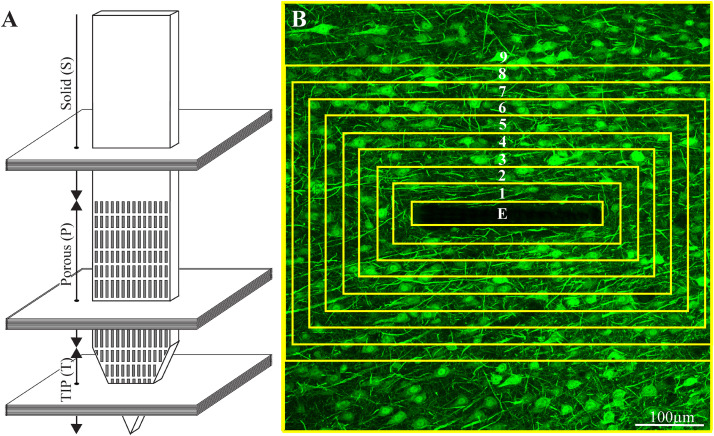
Schematic illustration of an implanted part of the perforated polyimide platform and the orientation of 40 μm thick horizontal cryostat slices along with paraformaldehyde fixed cortical tissue around it. **(A)** A number of cortical slices were prepared from the platform tip (T), perforated (P) and solid (S) parts. Consecutive confocal microscope optical sections were grabbed from the immunolabeled slices at intervals of 1 μm to prepare maximal projection images of the sectioned platform and the tissue surrounding it. **(B)** The integrated immuno-fluorescent intensity within the electrode (central yellow rectangle) and within nine, 25 μm wide centripetal shells around it were measured and processed to establish the normalized fluorescent intensity level (NFI) of a given cell type at a given distance around the electrode. The image in (B) depicts neuron cell bodies and neurites labeled by NeuN and NF, respectively.

Typically, 15-30 confocal slices were acquired, with a vertical spacing of 1μm. Image stacks were acquired from three regions of the electrode: tip (T), porous (P), and solid (S) (Figure 3).

### Image Processing, Analysis, and Statistics

The image processing was implemented using the Fiji distribution of ImageJ (Schindelin et al., 2012; Schneider et al., 2012), as follows. A maximum intensity projection image was created using 10 consecutive optical sections from each of the 40μm thick brain slices that was prepared. A rolling ball filter with a radius of fifty pixels was applied to the maximum intensity projection images to remove the background. This was implemented using the Subtract Background function in ImageJ. A region of interest (ROI) that defines the electrode was manually created. A set of concentric ROIs was automatically created around the electrode, where each ROI had a width of 25 μm, and the average intensity of the fluorescence signal was measured within each of these areas of interest (Figure 3). That same concentric ROI set was used on the corresponding control section in which there was no electrode, taken > 300μm away from the electrode. The normalized fluorescence distribution maps were created by dividing the value of the mean gray level of a given ROI (shell) from the image with the electrode by the value from the corresponding ROI in the control.

Average fluorescence values characterizing the FBR in space and time were measured and calculated from cortical brain slices prepared from 2 to 7 hemispheres/experimental points. Each brain hemisphere was used to prepare 1 to 5 tissue slices from each one of the three electrode segments (T, P, and S, Figure 3A). Each slice was used to prepare a single maximum projection image generated from 10 consecutive optical sections. The sample size of the immunohistological sections of the freely-flouting and fixed-to-the-skull platforms is given in Supplementary Table S1. Significant statistical differences between the Normalized Fluorescent Intensity (NFI) values at different distances from the implant and at different points in time were determined by a t-test for two samples assuming unequal variances. For all tests, a *P* value < 0.05 indicated a statistically significant difference (Supplementary Table S2).

Since the tissue for cross cryosections could not always be aligned perfectly, the apparent width of the PI platforms in the projected images may appear to be larger than 16 μm. As a result of the platform tilt, the asymmetric cell and nucleus distributions around the electrode were sometimes observed.

### Electron Microscopy

For TEM imaging of the glutaraldehyde/paraformaldehyde fixed tissue along with the PI, MEA platform implants were sliced by a Leica VT1000S Vibratome using a ceramic blades (Campden Instruments Ltd.) into 200 μm thick horizontal sections. The slices were deposited in 24 well plates with PBS.

After 8 washes with 0.1 M cacodylate buffer at pH 7.4 (Sigma Aldrich) the tissue was post fixed by 1% osmium tetroxide (Electron Microscopy Sciences) and 0.6% K3Fe(CN)_6_ in a 0.1 M cacodylate buffer for 1 hr. at room temperature. The slices were then washed again in a 0.1 M cacodylate buffer and dehydrated by a series of increasing concentrations of ethanol solutions of 10%, 25%, 50%, 75%, 90%, 96%, 100%, 100%. Finally, the slices were embedded in Agar 100 (Agar Scientific). The embedded preparation was then thin-sectioned and observed using a TEM Tecnai 12 microscope at 100 kV.

## Results

### Probe Design and Fabrication

The main design principles for the implants fabricated here were to produce potentially scalable MEA platforms of sufficient stiffness needed to implant the device without buckling, and importantly to be thin sectioned along with the surrounding tissue to enable immunohistological and electron microscope analysis. The recording MEA platforms were designed to enable wireless electrophysiological recordings from freely behaving rats. This was achieved by constructing an implant backbone using polyimide (PI). PI Young’s modulus is 2.5 GP and was experimentally shown to be compatible with brain tissue. Although other materials such as Parylene-C (Hassler et al., 2010, 2011), PDMS (Chou et al., 2013) and SU-8 (Rubehn and Stieglitz, 2010) with a lower Young modulus could be used, it was experimentally demonstrated that these do not significantly attenuate the FBR. Importantly, a number of laboratories provided experimental precedent indicating that implanted PI platforms can be thin sectioned along with the surrounding brain tissue for histological studies (Mercanzini et al., 2007, 2008; Richter et al., 2013; Xie Y. et al., 2014; Boehler et al., 2017). To optimize the integration of the implant with the brain tissue we fabricated a perforated PI-based MEA platform (PPMP) (Figure 1). The microarchitecture used was inspired by the “critical surface area hypothesis” described in Seymour and Kipke (2007) and Skousen et al. (2011) who demonstrated, that the FBR induced by a solid-shank silicon MEA platform was more severe than that caused by a lattice architecture. It was suggested that the perforation of the platform allows diffusion of molecules across the platform and growth of cells across the platform. In translating these concepts into a detailed design and fabrication, we produced and tested a number of PI platforms to best trade off: (1) the platform’s thickness and width, which defined the ability to implant the platform without buckling and the number of gold interconnects that could be embedded in the PI platform. (2) The size and density of the perforations that can also limit the number of interconnects and width. Three types of platforms were fabricated and used: (a) functional PPMPs, (b) Fixed-to-the-skull dummy implants and (c), free floating dummy PPMPs (Figure 1). The dummy PPMPs were used for the immunohistological studies. The fixed-to- the-skull platforms were identical to the functional PPMPs except for the lack of an output pad (Figure 1). As these implants were strongly fixed to the skull (Figure 2) they mechanically imitated the functional PPMPs. The free-floating dummy platforms were implanted without being fixed to the skull (Figure 2).

### Immunohistology of the Interface Between the Brain Tissue and the Implants

For economic and technical reasons related to the cost and low probability of successful cryosectioning of functional PPMP implants along with the skull and the screws that hold the implant without damaging the surrounding tissues, we used floating and fixed-to-the-skull dummy platforms (Figures 2C,D) rather than functional PPMPs for the immunohistological studies. Sections of the implants along with the surrounding tissue were examined immunohistologically 1 hour, 1 day, 3 days, 1, 2, 4, and 8 weeks after implantation (for sample sizes see Supplementary Table S1 and for statistical significance see Supplementary Table S2). A small number of months old functional PPMPs implants from which recordings were made were successfully processed for immunohistology and examined (Supplementary Figure S2).

Crucially, in contrast to classical silicon or metal based implants that have to be extracted from the brain tissue before sectioning, the perforated PI implants allowed us to section the implants along with the surrounding tissue and hence image the intricate structural relationships between the tissue and the implant. For the immunohistological studies, we prepared 40 μm thick horizontal slices of the cortex along with the implant covering its solid and perforated segments (Figure 3). For imaging, neuronal cell bodies and their neurites were labeled by NeuN and NF respectively (green), astrocytes by the glial fibrillary acidic protein (GFAP- red), and microglia by the calcium binding protein (Iba-1 - cyan). Since Iba-1 indistinguishably labels macrophages and microglia, it cannot be used to differentiate microglia from macrophages. To overcome this problem we co-labeled the cortical tissues by Iba-1 and TMEM119, an antibody that specifically recognizes rat microglia transmembrane proteins. This co-labeling unequivocally showed that from day 3 post implantation and onward the Iba-1 exclusively labeled microglia (Supplementary Figure S3). Proliferating cells were labeled by rabbit anti polyclonal KI67 (magenta). Nuclear DNA was labeled by 4′,6-diamidino-2-phenylindole (DAPI - yellow).

To improve the standard resolution of immunohistological labeled cell types, we took advantage of the characteristic distribution of the nuclei’s heterochromatin to identify the nuclei of the microglia, astrocytes and neurons (Supplementary Figure S4) (Garcia-Cabezas et al., 2016). Cells were identified as microglia, astrocytes, or neurons only when both the cytoplasmic and nuclear labeling criteria converged (Supplementary Figure S4).

All shown immuno-labeled images depict the maximal projection of ten consecutive optical sections grabbed at 1 μm intervals. To characterize the FBR along the different parts of the PPMPs, normalized fluorescent intensity (NFI) maps of each cell type, in nine centripetal, rectangular 25 μm thick shells around it were plotted.

**FIGURE 4:**
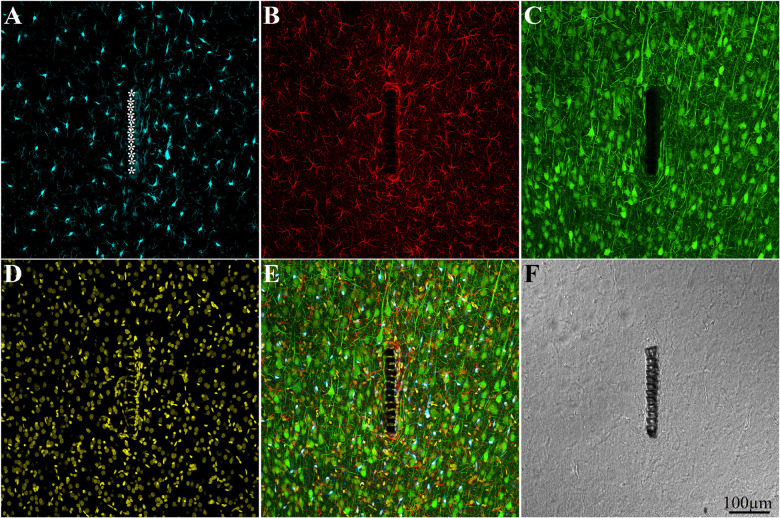
Confocal microscope images showing large field of view cross sections of cortical brain tissue along with the perforated segment of an implanted PPMP two weeks post implantation. Shown are: microglia around the implant marked by asterisks **(A)**, astrocytes **(B)**, neurons and neurites **(C)**, cell nuclei **(D)**, a merged image of **A–D (E)**, and a light microscope image of the implanted MEA platform and the surrounding tissue **(F)**.

To examine the contribution of the porous microarchitecture of the platform, NFI maps were prepared for the three different segments along the platform: the tapering perforated tip, the 280 μm wide perforated shank and the solid wide shank (Figure 3). The NFIs were analyzed (I), as a function of time after implantation (1 hour, 1 day, 3 days, 1, 2, 4, and 8 weeks) and (II), in relation to the mode of electrode implantation (free floating or fixed to the skull, Figure 2). Note that when using the NFI values to estimate changes in the spatiotemporal distribution patterns of microglia, astrocytes and neurons, the NFI integrates the cell body and branch fluorescence as well as the fluorescent intensity that corresponds to the different immunoreactivity levels of the labeled proteins which change during the transition from resting to reactive forms. The characteristic nuclei/cytoplasmic co- labeling provided accurate cell numbers when needed. An example of low magnification large field of view images of a perforated polyimide electrode and surrounding microglia, astrocytes, neurons and nuclei is depicted in Figure 4.

The main results and conclusions derived from the immunohistological analysis are as follows:

### Microglia

**FIGURE 5:**
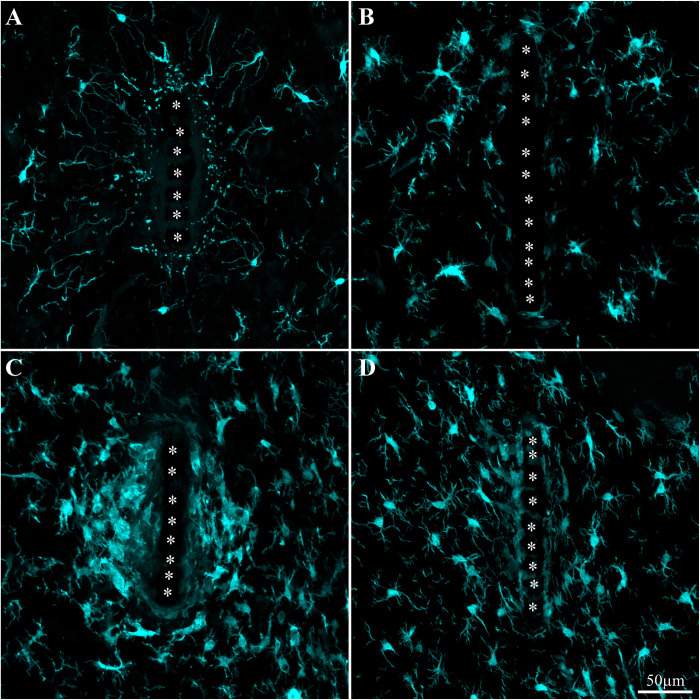
Activation and dynamics of microglia around implanted PPMP. The images depict cross- sections of a perforated platform along with Iba-1labeled microglia (cyan). For purposes of orientation, the PPMP is displayed at the center of the images. The solid PI “ridges” in between the pores (Figure 1) are labeled by white asterisks. **(A)** Cross- section of the PI platform along with the tissue around it prepared 1 h after the platform insertion reveals the presence of Iba-1labeled cytoplasmic particles in the vicinity and around the platform. These are most likely the remains of injured microglia that underwent a degenerative process. **(B)** One day post platform implantation, activated microglia characterized by short branches surround the platform. **(C)** Three days after the implantation the density of the amoeboid shaped activated microglia is increased. **(D)** One-week post implantation the density of the microglia declines and their morphology begins to regain the typical branched morphology of resting microglia. Note that microglia with weaker fluorescent intensity attach to the platform surface.

**FIGURE 6:**
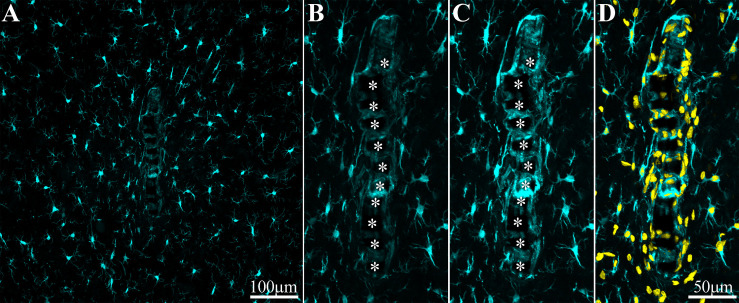
Adhesion of microglia to perforated implant surfaces and migration into the perforations. For purposes of orientation, the solid PI “ridges” in between the pores are labeled with white asterisks **(B,C)**. The images depict cross- sections of a perforated platform along with microglia (cyan) and their nuclei (Yellow, **D**) surrounding it two weeks post- platform implantation. At low magnifications **(A)**, conventionally used low numerical aperture objectives for mapping a large field of view of the FBR do not clearly reveal the faint Iba-1 fluorescent signal of microglia that adhere to the implant. Enlargement of the implant shown in **(A)** better reveals the faint Iba-1 fluorescent signal in contact and within the platform **(B)**. Selective contrast stretching of the Iba-1 fluorescent signal offline showed that in fact the pores and the platform surface were filled with microglia **(C)**. The density of microglia adhering to the platform surface and migrating into the pores becomes clear when the images of the identified microglia nuclei (yellow, and see Supplementary Figure S4) were merged with the images of the cytosol **(D)**.

**FIGURE 7:**
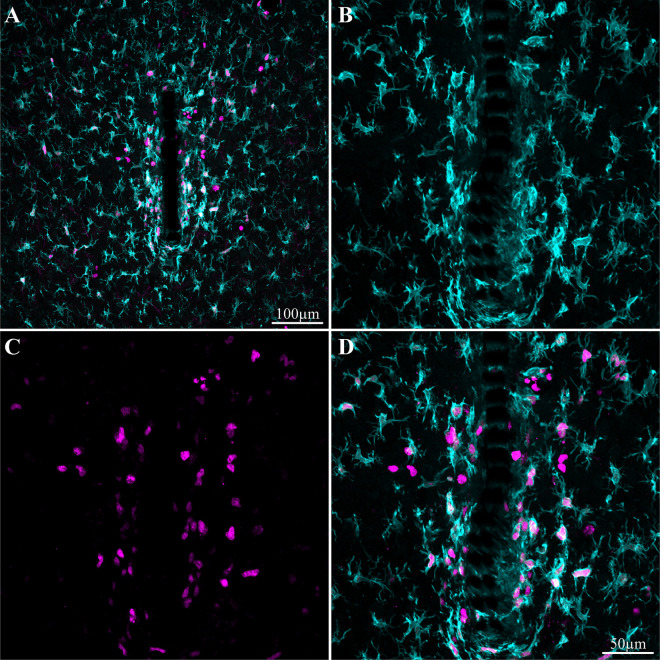
Induced microglia proliferation by the PPMP implant. The images were prepared 3 days after implantation. **(A)** Low magnification of co-labeled microglia by Iba-1 (cyan) and KI67 (magenta). Note that cell proliferation occurred mainly in the vicinity of the implant. **(B)** Enlargement of the implant (shown in **A**) and the microglia around it. **(C)** KI67 labeled proliferating cells. **(D)** Merged image of **B** and **C**.

One hour to one day post implantation the platform was surrounded by an acellular “shell” (edema Figures 5A,B). As documented previously by live two photon imaging (Eles et al., 2017) microglia at a distance of 50–100 μm away from the implant surface oriented and extended ramifying filopodia toward the implanted platform. Three days post implantation the shell around the implant filled up with dense activated microglia that differed from resting microglia by their amoeboid morphology. These microglia appeared to have stronger Iba-1 fluorescent intensity than the resting microglia that resided further away (∼ > 100μm) from the implant. At this stage, the activated microglia adhered to the PI surface and started migrating to occupy the platform pores (Figures 5, 6). Co-labeling of microglia by Iba-1 and KI67 that labels dividing nuclei showed that a fraction of the microglia around the implant but not microglia that resided ∼200μm away from it were dividing (Figure 7). Given that the half life time of adult resting microglia is 7.5–15 months (Fuger et al., 2017; Tay et al., 2017; Zhan et al., 2019) these observations imply that platform implantation changes the homeostatic molecular signature of the microglia to become inflammatory.

**FIGURE 8:**
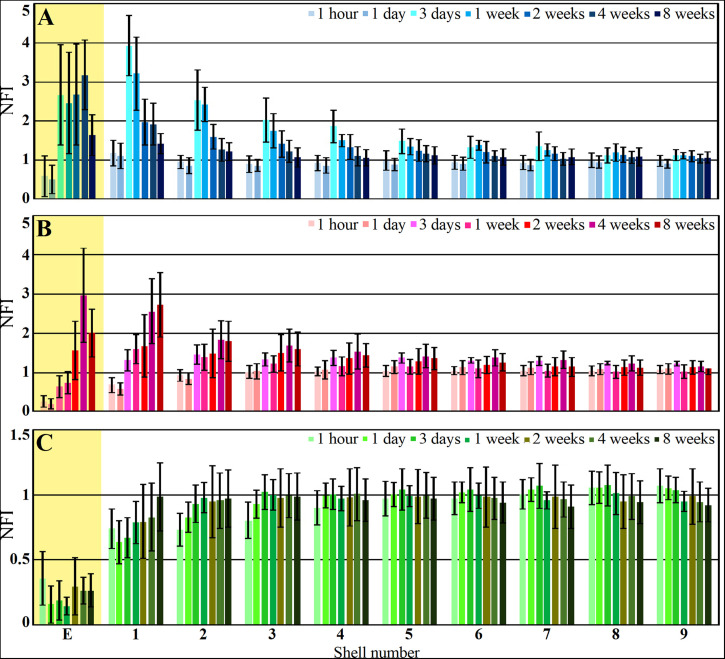
Histograms depicting the average Normalized Fluorescent Intensity (NFI) of microglia (**A**, blue), astrocytes (**B**, red) and neurons (**C**, green) within and around the platform’s perforated segments. The time post platform implantation is coded by the darkening of the column color as indicated by the legend on the right hand side of the histograms. The average NFI values within the platforms (E) are emphasized by the yellow background. The distance of the average NFI from the MEA platform is given by shell number. Each shell is 25μm wide (as illustrated in Figure 3). Vertical lines correspond to one standard deviation.

One week post implantation the rate of microglia division and migration subsided. Consistent with earlier studies, two weeks post implantation the density of activated microglia in the first shell around the implant began to decline (Figure 8 and Supplementary Figures S5, S6). Nevertheless, microglia with fainter Iba-1 fluorescent intensity remained adhering to the platform surfaces (Figures 5, 6). Note that it is difficult to detect the microglia that adhere to the PI platform since their Iba-1 fluorescent intensity is significantly fainter than the surrounding unattached microglia. To better illustrate this point we contrast stretched the Iba-1 fluorescent signal of the microglia in contact with the PI platform (Figure 6, compare B and C). Further to that, we merged to the Iba-1 image, with complimentary images of the DAPI labeled microglia nuclei (Figure 6D) that were prepared using the heterochromatin distribution pattern as detailed in Supplementary Figure S4.

It is plausible that in earlier studies using traditional large field of view low numerical objectives images these Iba-1 labeled cells were overlooked. In particular, the adhering microglia could have been missed since most studies extract the implant prior to sectioning the tissue for immunolabeling studies.

Although the microglia density (as defined by the NFI and counting) in the first shell around the implant was reduced, the density of the adhering microglia remained high (Figure 8 and Supplementary Figure S5).

Comparison of the microglia NFI distributions around free-floating and dummy implants fixed to the skull showed that the variability of the NFI values was larger in the fixed implants than in the free-floating platforms (Supplementary Figure S6). On average, the NFI values along some but not all segments of the platform were larger for the fixed platform. Overall, the average values showed that fixing the platform to the skull did not worsen the histological microglia FBR.

As discussed below, it is conceivable that the microglia that adhere to the PPMP rather than the thick multicellular encapsulating scar tissue, locally elevate the impedance between surrounding neurons and the electrodes, and thus diminish or even totally block the recorded FP amplitude.

### Astrocytes

**FIGURE 9:**
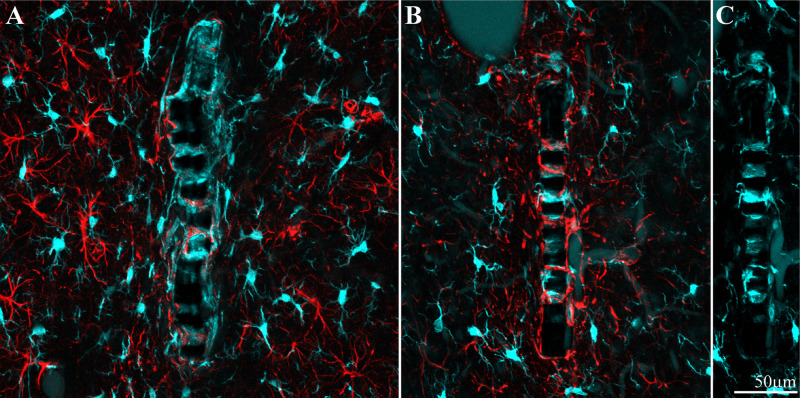
Different distribution patterns of microglia and astrocytes around and within an implant. **(A)** Two weeks and **(B)**, 8 weeks after implantation. In both images the platform surface is occupied by adhering cells in **(A)** mainly by microglia (cyan) and in **(B)** mainly by astrocytes (red). To reveal the presence of adhering microglia in **(B)**, the Iba-1 fluorescent signal of the adhering microglia was stretched **(C)**.

As described in earlier publications, astrocyte activation by cortical implants is delayed by approximately one week in respect to the activation of the microglia (Figures 8, 9 and Supplementary Figures S5, S6). Only two weeks after implantation, a moderate number of astrocytic branches were seen to adhere to the implant surface and extended into its perforations. It is worth emphasizing that the spatial resolution of confocal microscope imaging is insufficient to unequivocally determine whether microglia interface between the PI surfaces and the astrocytes or whether the astrocytes directly adhere to the PI platform. At two weeks post implantation the maximal astrocyte NFI levels within the first and second shells (0–25, 25–50 μm, respectively) around the platform tip and the perforated segments was ≤ 2 (Figure 8). This is a moderate value compared to similar studies that have recorded NFI levels ≥ 3 around solid platforms (for example, (Harris et al., 2011)). In contrast to the microglia, the astrocytes NFI level continued to increase and at weeks 4 and 8 post implantation reached NFI values of 2.5 and 2.7 (Figure 8, shell 1). Co-labeling of the astrocytes by GFAP and KI67 revealed sporadic astrocyte mitosis in the brain parenchyma but not in the in shells 1–3 around the implant. Unlike the microglia, astrocyte cell bodies were not observed to migrate into the platform pores at any point in time.

Interestingly, unlike the microglia the astrocyte NFI level did not diminish from the maximal value within or around the perforated PI platform, during the two month period post implantation (Figure 8). Although astrocyte activation is delayed in respect to the microglia (Figure 8), statistical analysis showed that both microglia and astrocytes were activated significantly in shells 1–5 (0–125 μm from the implant) in respect to control NFI levels two weeks after implantation (for microglia shells 1–5, *P* = 3.3e^–7^ – 0.023 and for astrocytes *P* = 0.00042 – 0.01854), but were not activated in shells 6–9, 125–200 μm further away (for microglia and for astrocytes *P* > 0.05).

In contrast to the microglia, the density of the astrocyte branches increased with time in the perforated parts of the platform but remained at the control level along the solid part (Supplementary Figure S5). Comparison of the astrocyte density distributions around the floating and fixed-to-the-skull implants showed that tethering did not worsen the astrocyte’s NFI with respect to the floating implants (Supplementary Figure S6).

### Neurons

**FIGURE 10:**
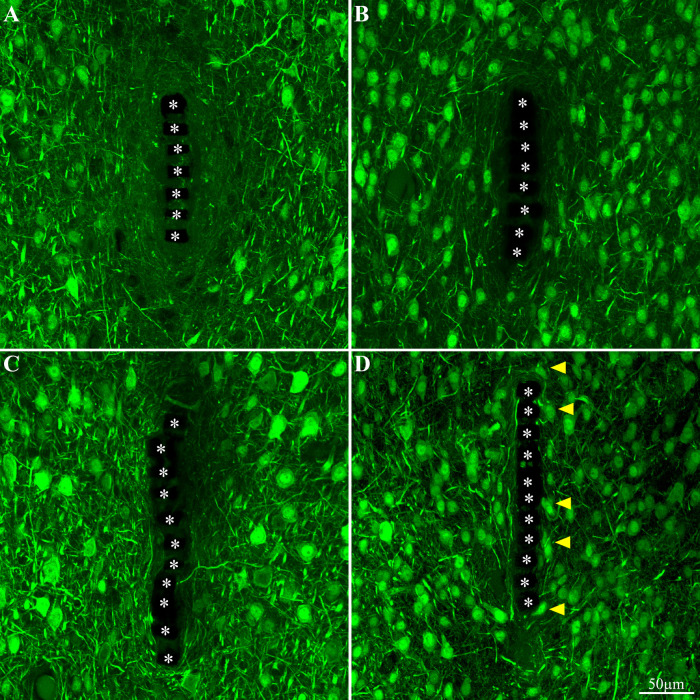
Neuron cell bodies and neurites labeled by NeuN and NF, at different time points post implant insertions. Cross -section of the PI implant at the perforated platform level along with the neurons around it prepared one hour after insertion **(A)**, 3 days, 2 and 8 weeks (**B–D**, respectively) after platform implantation. The perforations of the PI platform are indicated by the asterisks. The yellow arrow heads indicate neuronal cell bodies adjacent to the platforms surface.

Immediately after PPMP implantation and thereafter for 8 weeks the neuronal NFI (NeuN and NF combined) intensity within the perforated platforms domain remained low in the range of 0.14–0.36. As this level of NFI was observed in the perforated and solid regions of the implant one hour and a day after implantation, it most likely reflects auto-fluorescence of the platform’s PI backbone (Figure 10). Occasionally, neuronal cell bodies appeared to occupy a perforation. Immediate damage caused by the platform implantation to the neuronal population was detected within the first, second and third shells around the implant (0–75 μm) in which the NFI was reduced to 0.74 ± 0.15 in the first shell, 0.73 ± 0.13 in the second and 0.8 ± 0.14 in the third. With time, the NFI levels recovered to control in these shells. This recovery initially reflected regrowth of neurites toward the platform (Figure 10C). Later on, neuronal cell bodies were also found to occupy shells 1, 2 and 3. This observation is consistent with earlier reports suggesting that neurons migrate toward ultra-small and flexible cortical implants (Luan et al., 2017; Yang et al., 2019).

Comparison of the neuronal NFI intensity and distribution of fixed and free-floating implants over the implant surfaces and in time did not show any significant differences (Supplementary Figure S6).

### Transmission Electron Microscope Analysis of the Tissue Implant Interface

**FIGURE 11:**
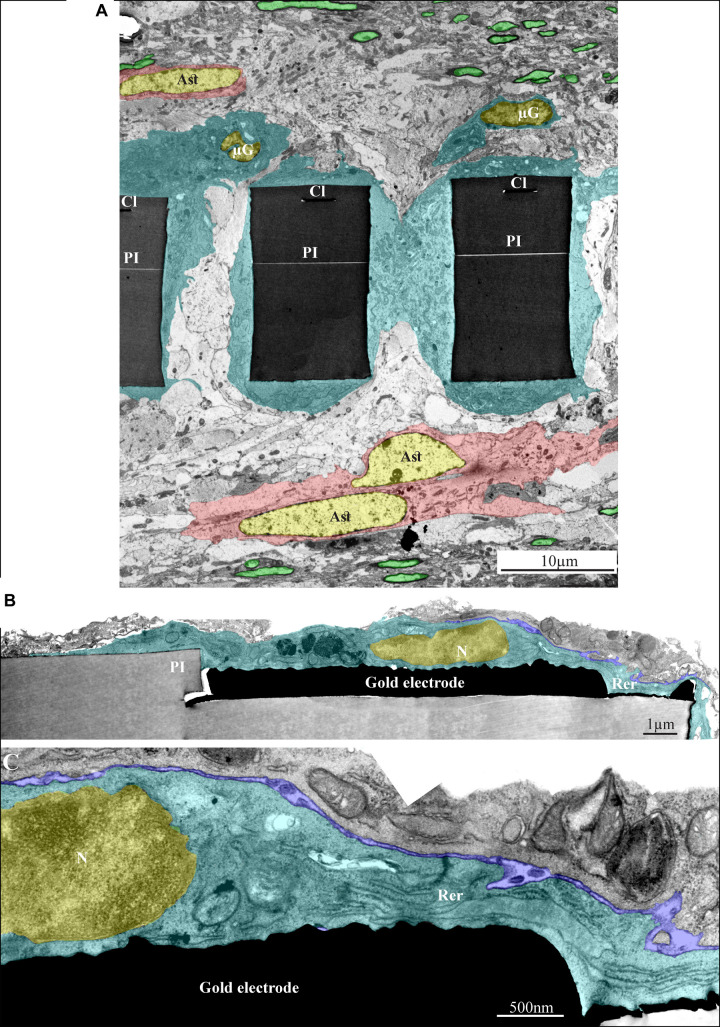
Ultrastructural integration of microglia and astrocytes with a perforated polyimide implant. **(A)** A low magnification cross-section of an implanted PPMP through the perforated segment. The PI “ribs” are encompassed by microglia (dark cytoplasm marked by cyan and typical microglia nuclei - μG marked yellow) and astrocytes (cytoplasm marked pink, and astrocyte nuclei - Ast yellow). **(B,C)** high magnification of microglia (cyan) adheres to a gold electrode and PI. Note the typical nuclear chromatin distribution of the microglia (yellow) and the lack of obvious extracellular space between the microglia membrane, the gold electrode and the PI surfaces. Extracellular space between the microglia and nearby cell is marked in blue. The images were prepared 14 days after the PPMP implantation. PI- polyimide, N - nucleus, RER- rough endoplasmic reticulum, Cl -conducting line, μG- microglia, Ast- astrocyte. Note an unmarked copy of this figure is presented as Supplementary Figure S7.

**FIGURE 12:**
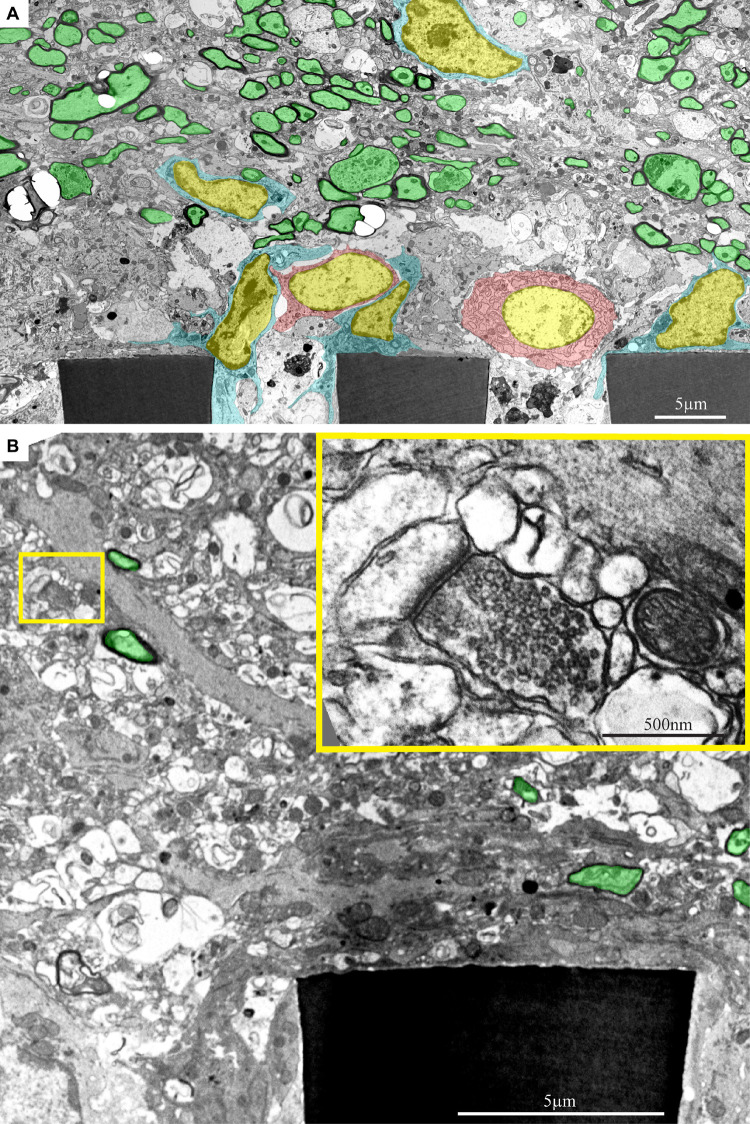
Ultrastructural images depicting the presence of myelinated fibers and synaptic structures within a distance of approximately 20 μm from the PPMPs surface. **(A)** A low magnification cross-section showing part of three polyimide “ribs” (PI) of an implanted PPMP. Microglia (cyan) and astrocyte cell bodies (pink) form contact and occupy the space around the PI ribs. Further away, at a distance of approximately 20 μm, the regenerating cortical tissue contains many profiles of myelinated axons (green). **(B)** A low magnification cross-section showing a part of a polyimide “rib” (PI) of an implanted PPMP. The “rib” is encompassed by a dark cytoplasm. A synaptic structure (yellow rectangle) composed of a presynaptic terminal field with synaptic vesicles and a post synaptic element depicted by it’s typical post synaptic density is located at a distance of approximately 10 μm from the PPMP surface. The images were prepared 14 days after the PPMP implantation. Note an unmarked copy of this figure is presented as Supplementary Figure S8.

As shown above, the use of a polyimide based implant that can be thin sectioned along with the tissue around it made it possible to improve the resolution of the interfaces formed between the surrounding parenchyma and the PPMP. Nevertheless, the practical resolution of fluorescent or confocal microscopes is insufficient to examine the ultrastructural relationships between different cell types and the implant. We therefore turned to use transmission electron microscopy (TEM) (Figures 11, 12 and Supplementary Figures S7, S8). The shown images and conclusions genuinely represent transmission electron microscope imaging of over 50 sections prepared from 11 different PPMPs implants.

Based on the characteristic chromatin distribution patterns (Supplementary Figure S4 and Garcia-Cabezas et al., 2016), we were able to identify the cell bodies of the microglia, astrocytes, and neurons in TEM sections. The quality of glutaraldehyde/paraformaldehyde brain fixation along with the implanted PPMPs for TEM processing and the preservation of the subcellular organelles including mitochondria, endoplasmic reticulum, synaptic vesicles, post synaptic densities and myelin were of good quality and permitted the identification of certain cellular extensions. Note that the observed extracellular spaces between the various cell types themselves and between them and the PPMPs surfaces reflected a documented shrinkage artifact of approximately 20% (Korogod et al., 2015). Despite the artifact and with full awareness of it, the fixation protocol used here is the most extensively used method nowadays. To overcome the chemical fixation artifacts a cryo-fixation method can be used (Korogod et al., 2015). Unfortunately, this procedure cannot be applied to cortical tissue implanted by a MEA platform without drastically damaging the structural relationships between the tissue and the implant.

As the overall spatial relationships between the PPMP and the cells around it are not altered by the fixation, and being fully aware of its limitations, we can conclude with certainty that microglia directly and strongly adhere to the implant. Consistent with this and the fact that the volume of the PPMPs is not altered by the fixatives, we observed that the fixation protocol often led to tears of the tissue around the implant. In many thin-sections cells adhering to the polyimide backbone of the implant or the gold electrodes themselves were torn apart, leaving one side of the cell attached to the platform and the other to the surrounding cells.

Since the range of the shrinkage factor is known, the genuine extracellular cleft formed between the adhering microglia plasma membrane and the implant surface can be estimated. Based on earlier *in vitro* studies of the extracellular clefts formed between cultured cells (in particular neurons) and solid silicone oxide substrates or substrate integrated electrodes, it is reasonable to assume that a cleft width in the range of 30–40 nm is formed between the microglia and the PPMP surfaces (Hai et al., 2009; Korogod et al., 2015; Ojovan et al., 2015; Shmoel et al., 2016). The seal resistance formed by such clefts (in parallel to the resistance to the ground) is likely to impede the current generated by nearby neurons from reaching the electrodes. The observations that the recording qualities and yield of the PPMPs deteriorated are consistent with the formation of the adhering microglia seal (see section “Discussion”).

In line with the immunohistological images of the Iba-1 labeled microglia cell bodies, TEM images revealed that the microglia directly adhere to the PI and the gold electrode surfaces and extend into the platform pores (Figures 11, 12 and Supplementary Figures S7, S8). The adhering microglia (as identified by the typical heterochromatin distribution), were characterized by dark cytoplasm with respect to other cell types and were enriched with smooth and rough endoplasmic reticula (Figure 11 and Supplementary Figure S7). Two weeks after implantation, the cytoplasm of the adhering microglia appeared to form a continuous encapsulation around the implant. At this point in time myelinated axonal profiles, and chemical synapses as identified by their typical post- synaptic densities and presynaptic vesicles were observed at distances of approximately 20 micrometers from the PI platform and up. Neuronal cell bodies (identified by their typical nuclei) were mostly observed at larger distances from the platform.

The observations described above (Figure 12 and Supplementary Figure S8) imply that regenerative processes such as neurites outgrowth myelination and synaptogenesis in the vicinity of the PPMP implant are taking place. Nonetheless, the microglia and possibly astrocytes that adhere to the implant surfaces prevent neurons from forming direct contact with the electrodes or the PI substrates.

### General Recording Characteristics

According to classical concepts, the spatiotemporal distribution patterns of microglia, astrocytes, neurons and other cell types that form the FBR are expected to predict the electrophysiological recording quality of a given implant (see section “Introduction”). Nonetheless, increasing numbers of studies emphasize that this is not the case and that in fact the histological severity of the FBR does not correlate well with the electrophysiological performance of recording implants (Kozai et al., 2014a, b, 2015a, b; McCreery et al., 2016; Du et al., 2017; Salatino et al., 2017a; Michelson et al., 2018).

**FIGURE 13:**
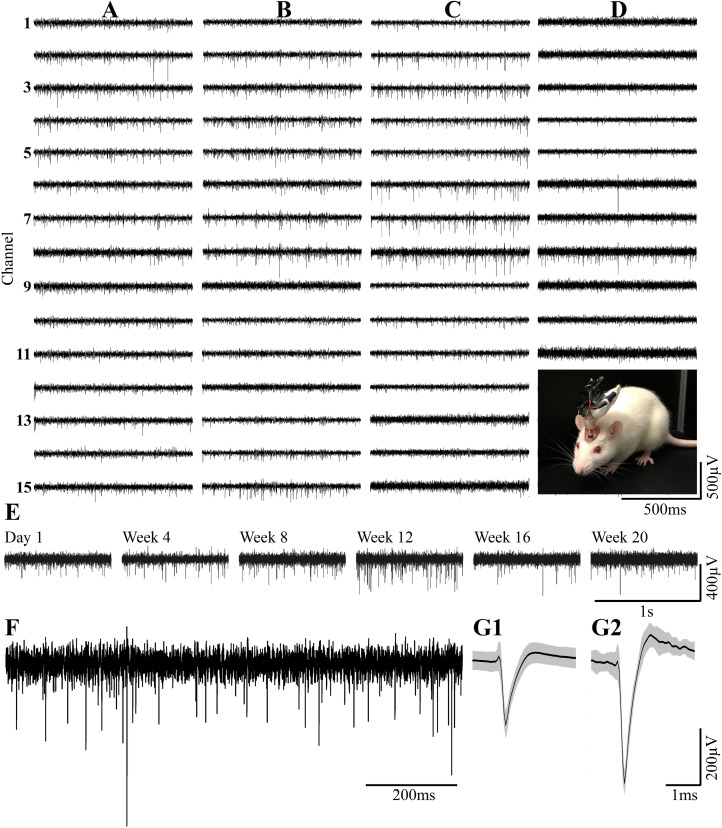
Examples of continuous long term wireless recordings over the course of 20 consecutive weeks after implantation show the spontaneous firing of FPs recorded by 15/15 microelectrodes. Spike sorting by the fully automatic spike-sorting implementation revealed that out of the 15 sensors implemented, 10 picked up one well-separated unit, 4 from two and 1 from 3 well-separated units. Column **(A)** One week post- implantation, **(B)** 1 month post- implantation, **(C)** 3 months and **(D)** 5 months post- implantation. Insert in **D** - the recordings are made from a freely moving rat implanted with a PPMP using a multichannel system wireless amplifier and battery attached to the implant by an omnetics connector. **(E)** Consecutive recordings of FPs by the same electrode on weeks 1, 4, 8, 12, 16 and 20 post-platform implantation. **(F)** An enlarged recording sweep at week 16. **(G1,G2)** Examples of average sorted FP (black line) and one standard deviation (gray background) from the sample sweep, shown in **(F)**.

**FIGURE 14:**
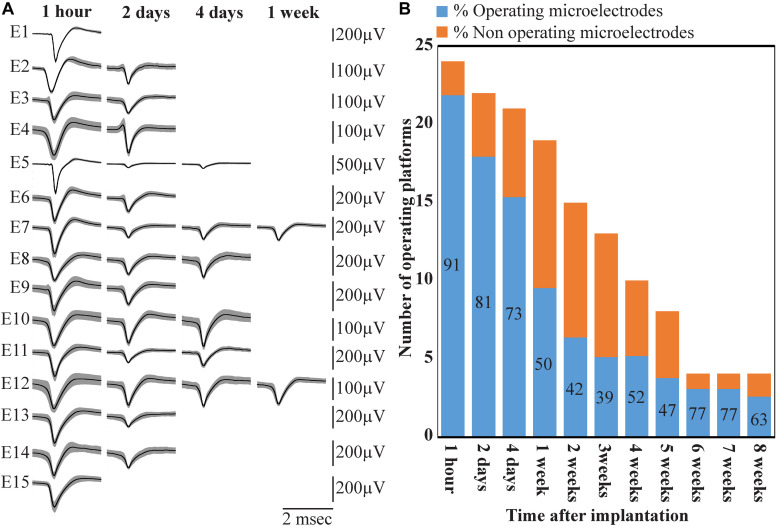
Deterioration of FP recordings yield over time. **(A)** An example of the deterioration of the averaged, sorted FPs recorded by a PPMP implant at one hour, 2, 4 and 7 days after platform implantation. **(B)** Out of the 24 implanted PPMPs (1 h after implantation) 91% of the electrodes were viable (blue). With time, the number of operative implants and the number of viable electrodes decreased. An implant is defined as operational if at least one electrode is viable. The percentage of viable electrodes (blue), at a given point in time, is defined as the number of viable electrodes out of the number of operating implants at that point in time X 15 electrodes/implant.

To gain insight to the relationships between the development of the inflammatory histological processes described above and the electrophysiological performance of implanted PPMPs we examined the PPMP’s recording performance over two months periods (Figures 13, 14). To that end 24 functional PPMPs (identical in all physical features to the dummy PPMPS used for the structural studies, Figure 1) were implanted into rat motor cortices as described in the Method section (Figures 1, 2A,B).

Wireless recordings were made online during platform insertion to help position the electrodes and thereafter the wireless recording from awake, freely moving rats were conducted an hour after implantation, two and four days after implantation. Thereafter recordings were continued once a week for 8 weeks (in a few cases the recordings continued for 8 months). The recording sessions were terminated when no spontaneous firing activity was recorded for two consecutive weeks. The average noise level of these electrodes was 20 ± 4 μV and the signal to noise ratio (SNR) ranged from 5 to 16. The largest FP recordings of 300–1100 μV were obtained one hour after implantation from all 24 PPMP implants with recordings from 91% of the total number of electrodes. The percentage of viable electrodes, at a given point in time, is defined as the number of viable electrodes out of the number of operating implants, times 15 electrodes/implant. An implant is defined as operating if at least one electrode is viable. Out of the 24 tested PPMPs four remained functional for over 8 weeks (Figure 14). Among these, in two platforms all 15 recording electrodes remained viable for the entire duration (Figure 14). In the majority of the experiments the FP amplitudes were attenuated to the range of 80–300 μV within the first week of implantation. In parallel, the number of operating implants and the percentage of viable electrodes dropped. Two days after implantations the number of operating implants dropped from 24 to 22 and the percentage of viable microelectrodes dropped from 91% to 80%. The number of operating implants and the percentage of viable electrodes continued to deteriorate as illustrated by the histogram of Figure 14. It is noted that between day two and 14 after implantation the number of operating implants dropped from 22 to 15 and the percentage of viable electrodes from 80% to 42%. This window of time temporally correlate with the migration, division and attachment of microglia to the PPMP’s surfaces (Figures 5, 6, 7). The decrease in the FP amplitudes was associated with increased electrode impedance from 1.5 ± 0.37 MΩ (at 1 KHz) to 2.96 ± 0.89. Of interest to note is the observation that a number of electrodes that ceased to record for a week spontaneously recovered to record FPs.

## Discussion

Pioneering studies designed to understand the mechanisms underlying the deterioration of extracellular field potentials recordings by cortical MEA implants over days after implantation have attributed this phenomenon to the initiation and perpetuation of local inflammatory processes by the implanted foreign body; i.e., the Foreign Body Response (for details see introduction). Meticulous attempts to establish causal relationships between the deterioration (as defined by the FP amplitudes, and the number of functioning electrodes) and the severity of the FBR (as defined by the density and distribution of microglia, astrocytes and neurons and other cell types) have failed (Kozai et al., 2014b, 2015b; McCreery et al., 2016; Du et al., 2017; Salatino et al., 2017a; Michelson et al., 2018). The present study shifts the focus of attention from the hypothesis that the FP amplitudes passively decrement across the resistance presented by the multicellular, multi-layered FBR scar to suggest that the abrupt decrement of the FPs occurs at the surface of the implant by microglia that adhere to form a seal at the implant surface. Uncovering this mechanism became feasible through the use of the PI based perforated implants and gold microelectrodes which made it possible to thin-section the implant along with the surrounding tissue for purposes of confocal and transmission electron microscopic analysis. This is in contrast to the classical studies in which implants had to be extracted before processing the brain tissues for immunohistological examination. Based on our observations, we suggest that microglia adhering to the implant surfaces form a high seal resistance over the electrodes and that this seal limits or even totally blocks the electrical coupling between the neurons and the electrodes. Because this seal is formed at the electrode surface this mechanism provides a potential explanation to why no correlation has been found between the dimensions and density of the FBR and recording qualities. If correct, future research to specifically mitigate the formation of the insulating glia could lead to improved recording quality and yield.

### Electrical Insulation of Implanted MEA Platform

Attenuation of FP amplitude at the surface of a MEA implant could occur by a number of mechanisms: (A) a “blocking” ion bi-layer formed at the metal electrode surface and the extracellular ionic solution of the brain (McAdams et al., 2006), (B) a self-assembled biofouling layers on the electrode surface (Sommakia et al., 2009, 2014a; Malaga et al., 2016), and (C) by increased impedance produced by cells adhering to the electrode surfaces as demonstrated for cochlear implants (Newbold et al., 2010, 2014). Based on our TEM and immunohistological observations it is conceivable that in cortical brain implants, microglia and possibly astrocyte that adhere directly to the electrode may play a dominant role in electrodes deterioration and subsequent insulation.

Despite the vast number of studies showing that microglia are the first cell line to be recruited by electrode implantation and that they release cytokines that activate cell cascades culminating in the characteristic histological structure of the FBR, the electrical insulation of implanted electrodes by adhering microglia has not explicitly been considered as a mechanism for reduction in FP amplitude. This is probably due to the fact that the microglia adhering to the implant surface are removed when the implants are extracted from the brain prior to fixation for histological examination, and the fact that the large field-of-view, low numerical aperture objectives conventionally used to characterize the FBR cannot detect adhering microglia. The possible presence of an insulating microglia is in line with live two-photon imaging studies of microglia in response to electrode implantation documented by the X. T. Cui group. In a series of studies, this group showed that within 10–12 h. post implantation, microglia start to migrate toward an implanted silicon probe, and within approximately 3 days of implantation almost 100% of the electrode is surrounded by microglia (Kozai et al., 2014a; Eles et al., 2017; Wellman and Kozai, 2018). Although the spatial resolution (especially on the Z axis) of live two-photon imaging is insufficient to determine the thickness of the extracellular cleft formed between the microglia and the electrodes, the rapid and directed migration of the microglia toward the implant surfaces constitutes the precondition for the microglia to be the first to recognize and adhere to the foreign implant. Our TEM images showed that the microglia directly interface and adhere to the entire length of the gold electrodes and even beyond it to the polyimide. Since the nature of thin sections prepared for TEM imaging precludes examination of the entire surface area of a given electrode, it is assumed that segments of the electrode surfaces might be glia free. Therefore it is reasonable to assume that different electrodes may reveal different levels of electrical coupling with the surrounding neurons. Because brain tissue shrinks after fixation for TEM processing, the thickness of the actual extracellular cleft formed between the microglia and the electrode surface cannot be precisely measured.

**FIGURE 15:**
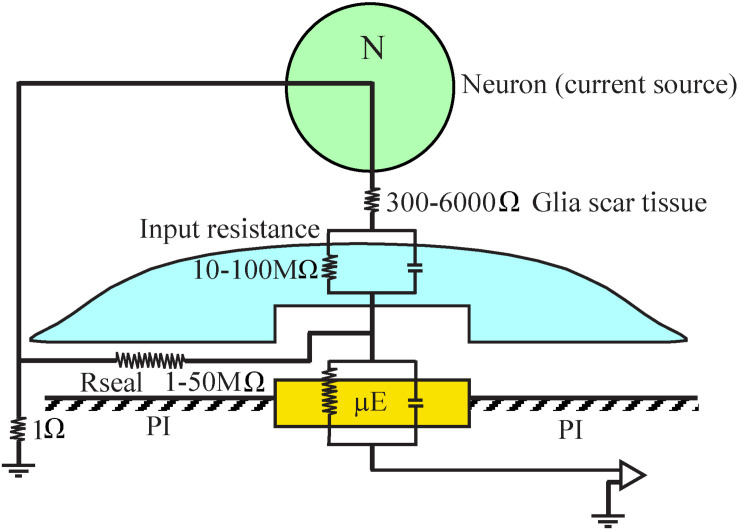
A minimal analog electrical circuit depicting the relationships between a current source - neuron (N, green) and an implanted microelectrode (μE yellow) to account for the abrupt attenuation of the FP amplitude by microglia (blue) that adheres to the microelectrode surface and the polyimide substrate around it (PI, gray). The configuration shows the current source (neuron), a resistor representing the scar tissue in series with the input resistance of a glia cell adhering to the electrode and the electrode impedance. The resistance formed by the extracellular space between the adhering cell (blue) and the microelectrode (yellow) is represented by the seal resistance (Rseal). The brain tissue resistance between the current source (neuron) and the ground is depicted on the left hand side. The given resistance values are orders of magnitude estimates from the literature. For additional information, see text.

An orders of magnitude approximation of the attenuation factor introduced by the adhering microglia can be obtained by examination of a passive analog electrical circuit of the electrode and the brain tissue (Figure 15). A minimal circuit depicting the configuration as shown below incorporates the following elements: (a) three resistors in series depicting the scar tissue in between the current source (neuron) and an adhering glia cell to the electrode, the input resistance of the microglia adhering to the electrode surface, and the electrode impedance. (b) the seal resistance (Rseal) formed by the “extracellular” cleft between the adhering glial cell membrane and the electrode surface, and (c), the resistance between the current sources (neurons) and the ground.

(a) The input resistance of resting microglia is in the range of 2–5 GΩ (Avignone et al., 2008; Schilling and Eder, 2015). This input resistance may be significantly reduced in conjunction with the functional state of the microglia (Eder, 1998, 2005, 2010; Kettenmann et al., 2011). Even if we assume that the input resistance of microglia that adhere to an electrode is reduced by one to two orders of magnitude, their bulk resistivity will be in the range of a hundred to ten MΩ. (b) It is difficult to estimate the value of the seal resistance formed between adhering glia and the electrode surface since fixation artifacts are known to reduce the CNS extracellular spaces (Korogod et al., 2015; Nicholson and Hrabetova, 2017). Assuming that a single microglial cell totally covers an electrode, and based on in- vitro studies showing that the cleft thickness formed between different cell types and artificial substrates ranges from 20 to 100 nm (Braun and Fromherz, 1998; Iwanaga et al., 2001; Straub et al., 2001; Lambacher and Fromherz, 2002; Brittinger and Fromherz, 2005; Gleixner and Fromherz, 2006; Wrobel et al., 2008), the estimated seal resistance for such a cleft would be in the range of a few to tens of MΩs (Weis and Fromherz, 1997; Buitenweg et al., 1998, 2002; Spira and Hai, 2013; Ojovan et al., 2015; Shmoel et al., 2016; Spira et al., 2019). (c) The estimated value of the parallel resistance between a neuron located close to the electrode and a ground located centimeters away is in the range of 300–6000 Ω, including an encapsulation layer (Turner et al., 1999; Szarowski et al., 2003; Moffitt and McIntyre, 2005; Grill and Mortimer, 2014) and in the range of 1–4 Ω in an intact brain (Logothetis et al., 2007).

In summary, the resistance along the current paths from the neuron to the electrode is substantially larger than the resistance on the current path from the neuron to the far ground. Since this latter resistance is substantially smaller than the first two, it is plausible that the current generated even by neurons positioned close to the sensing electrodes to which microglia adhere is attenuated by orders of magnitude and thus is practically not detected. The input resistance of adult astrocytes is in the range of single MΩs (Hanson et al., 2016) so that the theoretical attenuation factor of an adhering astrocyte may be somewhat lower.

These orders of magnitude estimates imply that FPs generated by neurons can be abruptly attenuated or blocked locally at the interfacing junction between the electrode and the tissue. Thus, we posit that the seal resistance formed by adhering glia to the electrodes rather than the resistance generated by multicellular encapsulating scar plays the dominant role in the deterioration and blockade of FP recordings by implants, independently of platform flexibility or dimensions.

Whereas it is conceivable that the model of abrupt FP attenuation by adhering microglia represents a common mechanism that can be generalized, implants made of different materials, having different sizes and shapes and implanted in different brain regions may elicit different inflammatory cell cascades. Obviously, neuron displacement, changes in neuronal excitability and synaptic connectivity around implanted electrodes contribute to the deterioration of the recorded FPs and yield.

Thus overall, the recording qualities and stability of MEA implants depend on a large number of cell biological and molecular factors that unfold in parallel and sequentially in time and space. Further examinations of the adhering microglia as downstream elements in the cascade of local inflammation are needed. This may facilitate the development of specific means to prevent the formation of the microglia sealing and thereby improve the recording qualities and stability of implanted MEA platforms.

## Data Availability Statement

All datasets presented in this study are included in the article/Supplementary Material.

## Ethics Statement

The animal study was reviewed and approved by Animal Care and use committee Faculty of Science, The Hebrew University of Jerusalem.

## Author Contributions

NS and S-HH designed and fabricated the perforated polyimide MEA platforms. MJ developed the platform implantation approach and with AS implanted the PI platforms. HE, AS, and MJ conducted the electrophysiological recording sessions. HE and WA-S prepared and analyzed the immunohistological sections. HE prepared the TEM materials and the figures. AW developed the software for image processing. IN supervised the analysis of the electrophysiological data. MS conceived, designed, supervised the project, and wrote the manuscript. All authors contributed to the article and approved the submitted version.

## Conflict of Interest

The authors declare that the research was conducted in the absence of any commercial or financial relationships that could be construed as a potential conflict of interest.
