# Supplementary material for: Immunohistological and Ultrastructural Study of the Inflammatory Response to Perforated Polyimide Cortical Implants: Mechanisms Underlying Deterioration of Electrophysiological Recording Quality
Source: Front Neurosci. 2020 Aug 31;14:926. doi: 10.3389/fnins.2020.00926 (PMC7489236; doi:10.3389/fnins.2020.00926)
Supplement: Supplementary file 1 [file Data_Sheet_1.pdf]

Supplementary material

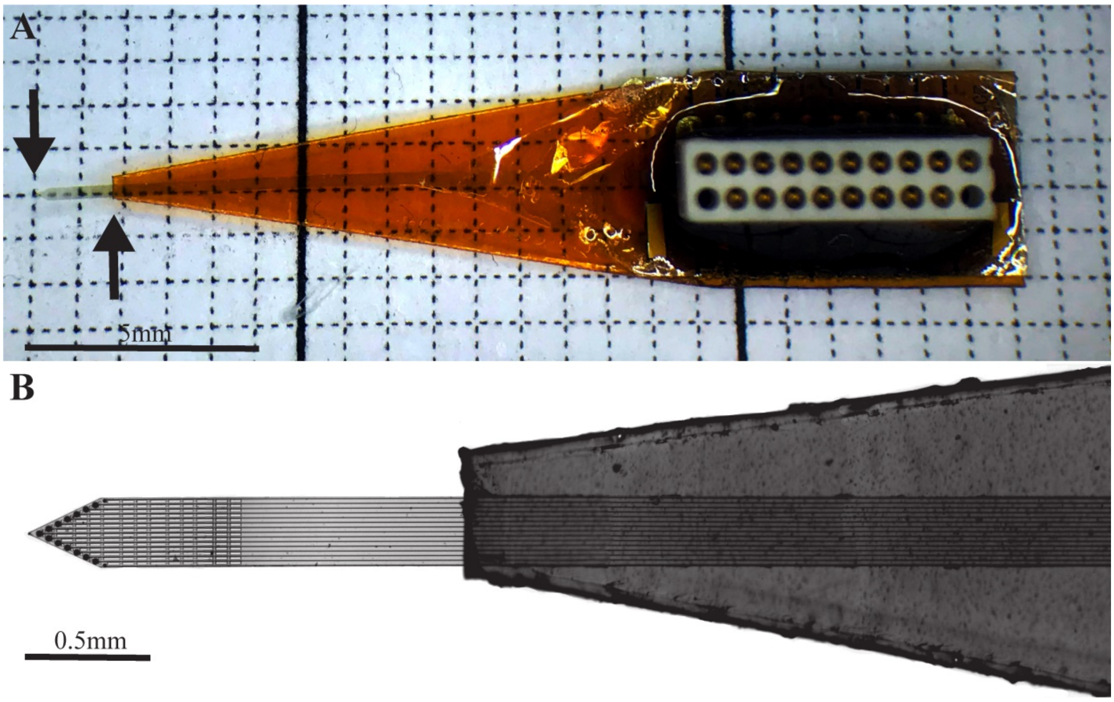

Supplementary Figure S1. Images of the Perforated Polyimide MEA Platform (PPMP) glued to a 300 µm thick kapton tape for mechanical stabilization. (A) Low magnification of a PPMP connected to an omnetics connector (right hand side) and glued to a kapton tape that narrows towards the tip of the platform (brown color). The kapton-free distal shank to be inserted into the brain is indicated by two arrows. (B) Large magnification of the distal segment of the PPMP glued to a kapton tape. Shown are the kapton-free distal PPMP shank, its tip and the most distal part of the kapton tape. Experimental testing confirmed that a 2 mm long kapton-free shank can be inserted through the pia into the cortex. When the pia is punctured prior to insertion a 3 mm long kapton-free shank can be inserted.

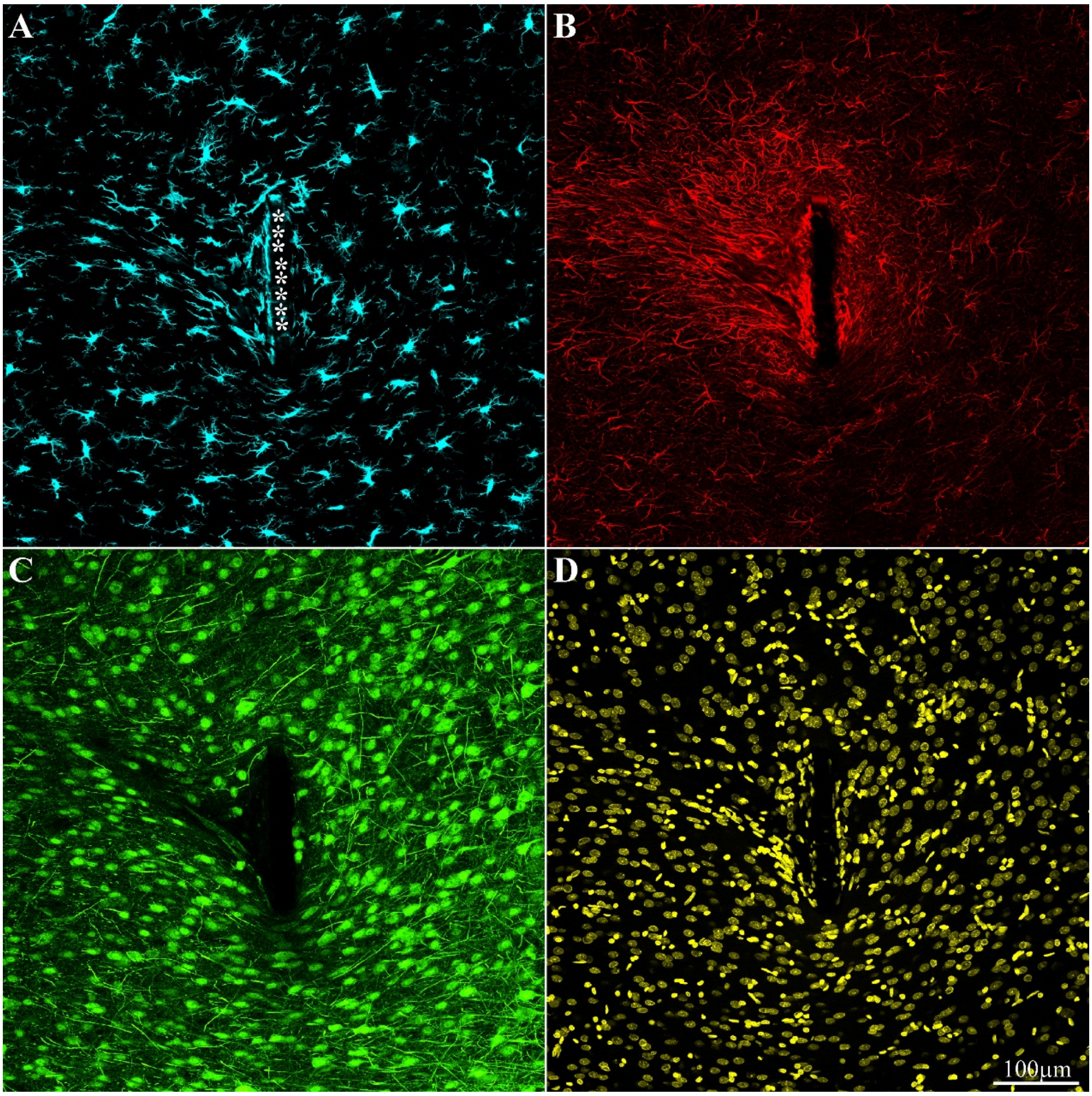

Supplementary Figure S2. Immunohistology of functional electrode, 22 weeks after implantation. Confocal microscope images showing cross sections of cortical brain tissue along with the perforated segment of a functional PPMP implanted for 22 weeks. Shown are: microglia around the implant marked by asterisks (A), astrocytes (B), neurons and neurites (C) and cells nuclei (D).

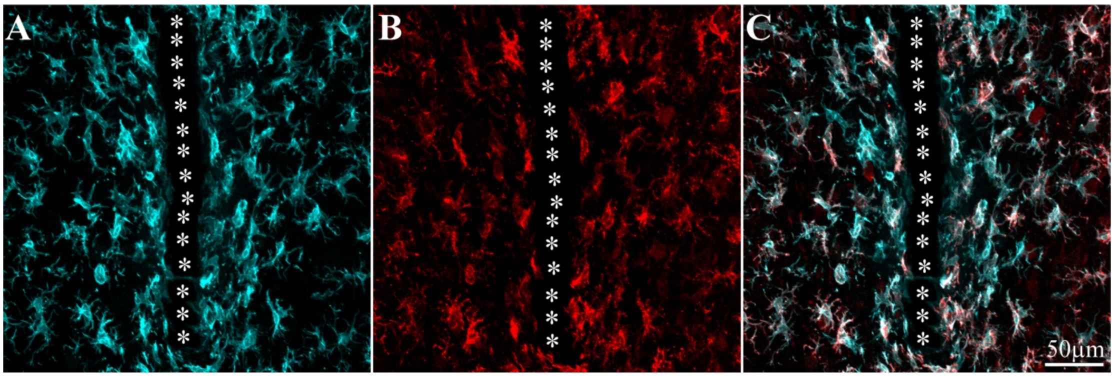

Supplementary Figure S3. Microglia co-labeled by Iba-1 and TMEM119, an antibody that specifically recognizes rat microglia transmembrane proteins. Confocal microscope images showing a cross section of a PPMP (asterisks) along with the surrounding microglia. (A) Iba-1, (B) TMEM119, (C) Merged image of A and B.

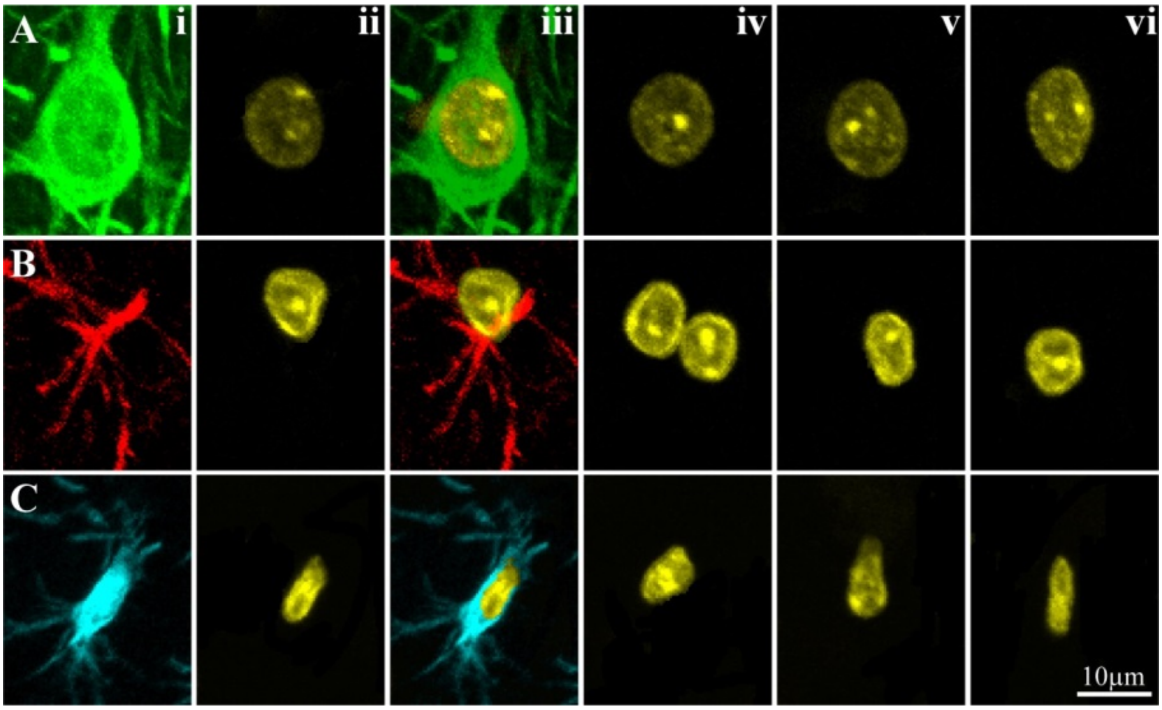

Supplementary Figure S4. Characteristic nuclear morphologies and heterochromatin distributions in neurons, astrocytes and microglia. Column I (rows A, B, and C) depicts the specific and characteristic immuno-labeling of a neuron (by NeuN and NF- green), astrocytes (GFAP- red) and microglia (Iba1-cyan). In column II, the DAPI labeled nuclei of the cells in column I are displayed. Column III (rows A, B and C) are merged images of the cells' cytoplasm and their corresponding nuclei as shown in columns I and II. The nuclei of neurons are characterized by round shaped "empty" appearing nucleus, a prominent nucleolus and a faint nuclear membrane. Additional examples of neuronal nuclei are shown in columns IV, V, and VI (row A) to document the range of their structural variability. Row B shows an astrocyte labeled by GFAP and its corresponding nucleus (II) and a merged cell nucleus image in (III). The characteristic feature of astrocyte nuclei is that in addition to having a round shape and a central nucleolus, the nuclear membrane is decorated by heterochromatin. Additional examples of astrocytes nuclei are shown in columns IV, V, and VI (row B) to document the range of their structural variability. Microglia nuclei (row C) are smaller than neuronal and astrocyte nuclei and are characterized by an oval shape and distributed puncta-like heterochromatin. Additional examples of microglia nuclei are shown in columns IV, V, and VI (row C) to document the range of their structural variability.

The significant differences in the nuclear shape and heterochromatin distribution of neurons, astrocytes and microglia enabled us to generate images that depict the distribution of one type of nuclei. To that end images of DAPI labeled cells were blown up, and all nuclei that do not show the characteristic heterochromatin distribution pattern of one cell type and were no co-labeled with the characteristic immuno-labeling of the cell type that was checked, for example microglia (Fig. 6D), were manually erased. To reduce the probably of erroneous misclassification in the identifications of microglia nuclei, the procedure was carried out independently by two trained specialists.

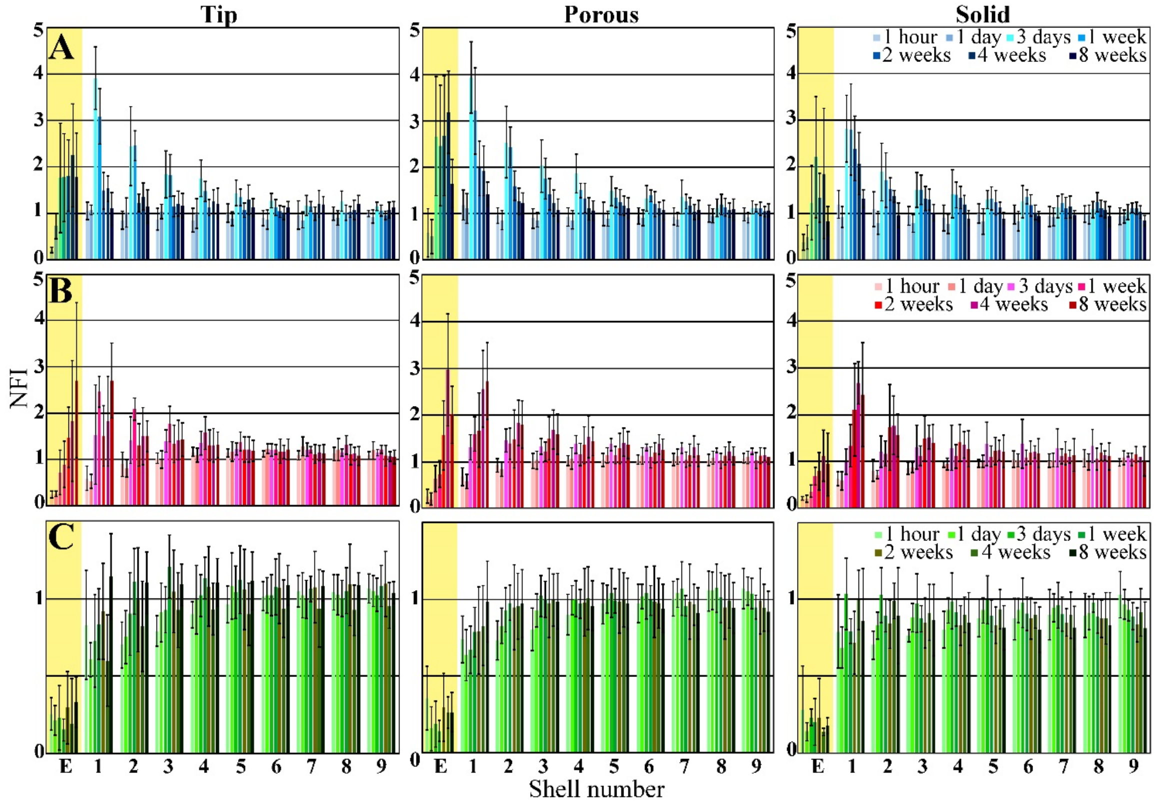

Supplementary Figure S5. Histograms depicting the average Normalized Fluorescent Intensity (NFI) of microglia (A, blue), astrocytes (B, red), neuron cell bodies and neurites (C, green) within and around the tip of the platform (left hand side column) the perforated segment (middle column) and the solid segment (right hand side column). Time post- platform implantation is coded by the darkening of the column color as indicated. The average NFI values within the platforms (E) are depicted by the yellow background. The distance of the average NFI from the perforated PI-MEA platform is given by shell number. Each shell is 25µm wide (as illustrated in figure 2). Vertical lines correspond to one standard deviation.

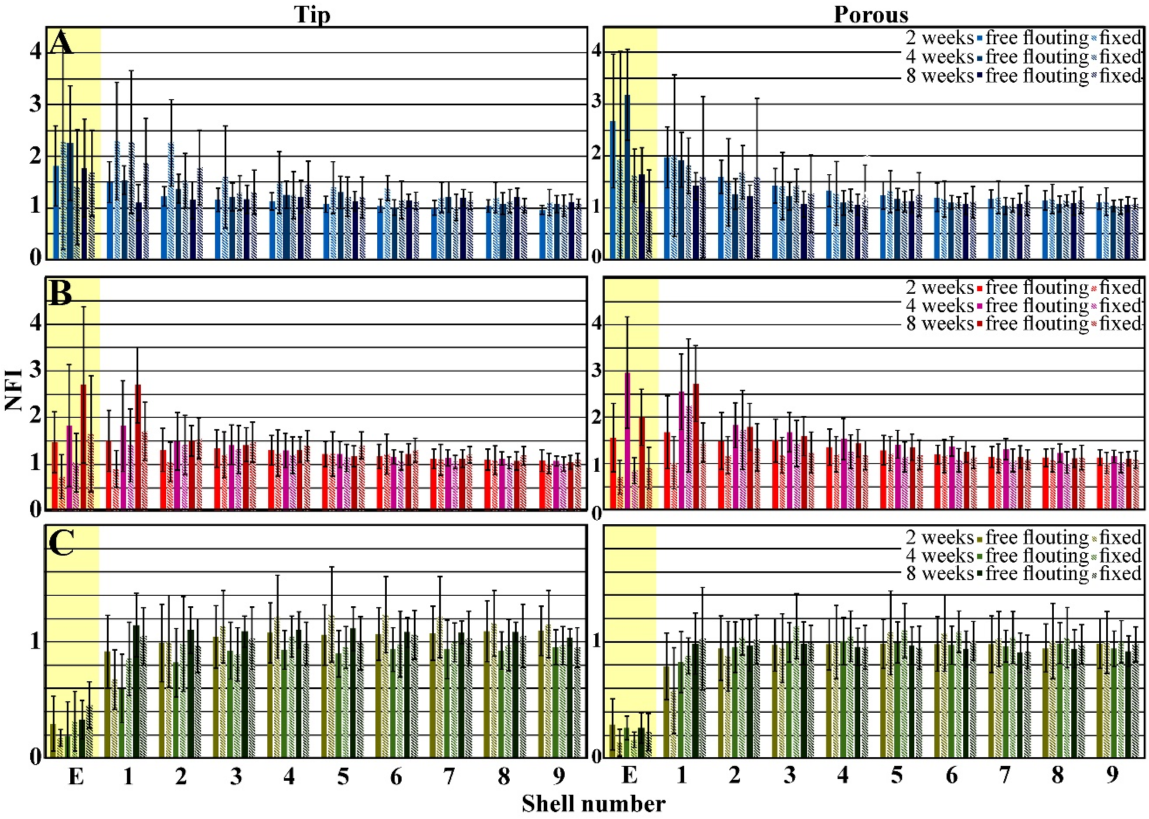

Supplementary Figure S6. **Histograms depicting the average NFI of microglia (A, blue), astrocytes (B, red), neuron cell bodies and neurites (C, green) within and around free-floating (smooth coloring of the columns) and fixed-to-the-skull platforms (chattered coloring) at the tip of the platform (left hand side column) and the perforated segment** (right column) at different time points and distances from the implanted platform. Time post- platform implantation is coded by the darkening of the column color as indicated. The average NFI values within the platforms (E) are depicted by the yellow background. The distance of the average NFI from the perforated PI-MEA platform is given by shell number. Each shell is 25µm wide (as illustrated in Figure 2). Vertical lines correspond to one standard deviation.

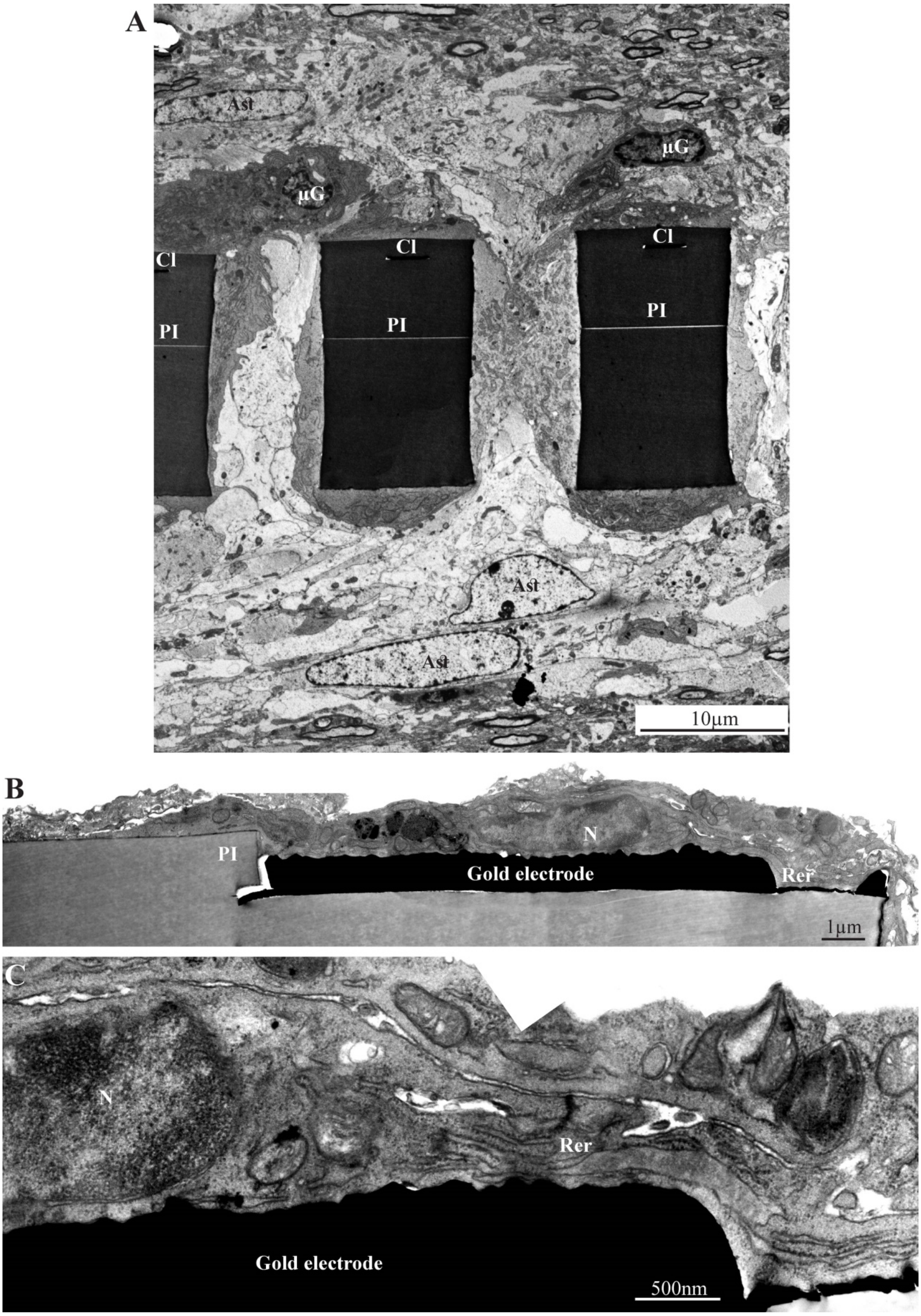

Supplementary Figure S7. Unmarked transmission electron micrograph shown in the main text as figure 11.

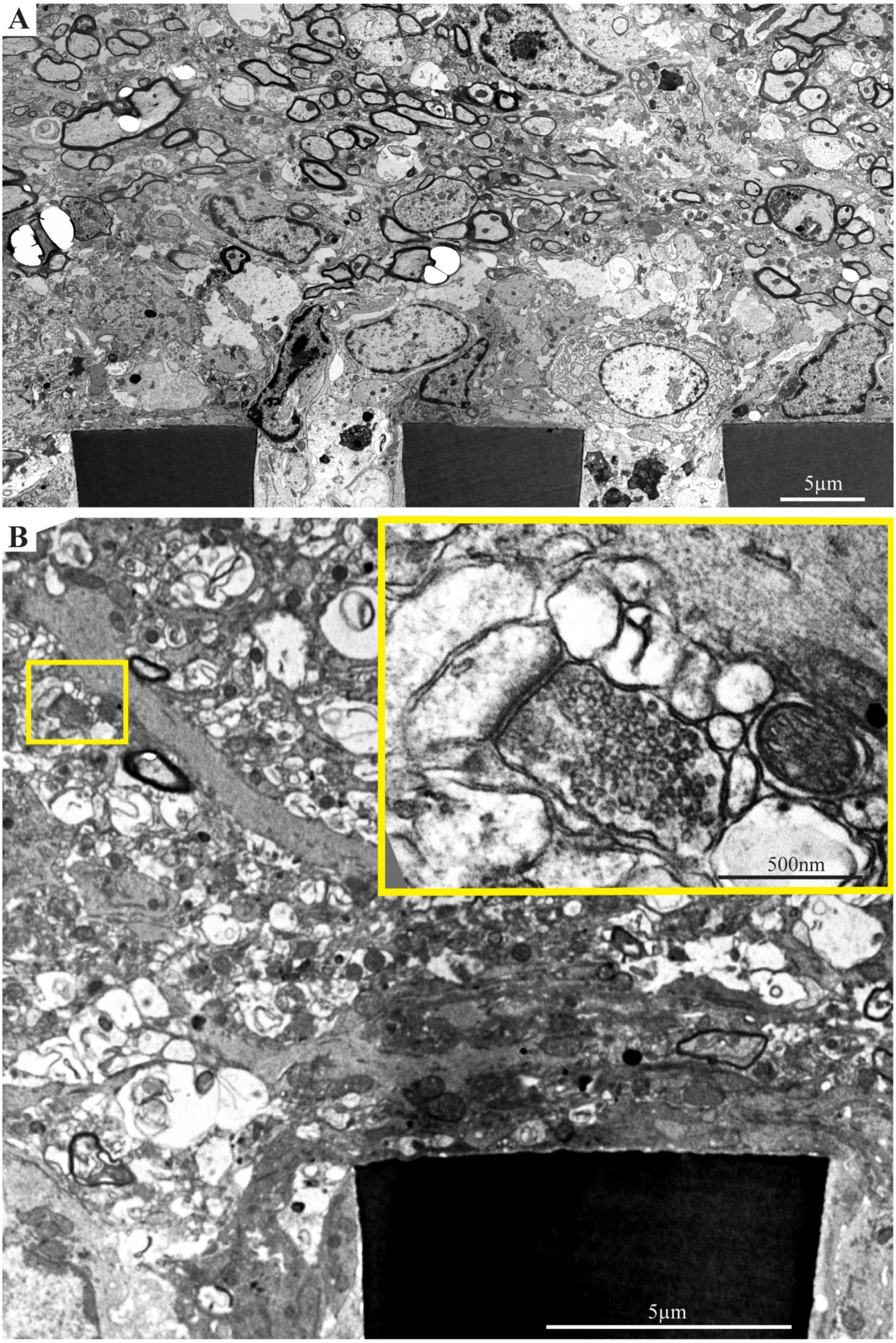

Supplementary Figure S8. Unmarked transmission electron micrograph shown in the main text as figure 12.

		Free floating implants							Fixed-to-skull implants		
**Time**		**1 hour**	**1 day**	**3 days**	**1 week**	**2 weeks**	**4 weeks**	**8 weeks**	**2 weeks**	**4 weeks**	**8 weeks**
**Microglia**	Tip	11/3	8/4	10/3	8/2	22 / 6	10/ 3	11/ 4	13 / 4	9/ 3	11/4
	Porous	21/4	20/4	10/3	13/4	21/ 6	13/ 3	18/ 4	14/ 4	12/ 3	14/4
	Solid	10/3	15/4	5/2	9/4	15/ 6	7 /2	11/ 4	4/ 1	5/ 2	0
**Astrocyes**	Tip	11/3	8/4	7/3	5/2	24 /7	10/ 3	11/ 4	13 /4	9/ 3	11/4
	Porous	19/4	18/4	6/3	6/4	25/ 7	13/ 3	18/ 4	14/ 4	12/ 3	15/4
	Solid	7/2	10/3	4/2	8/2	17/ 7	7 /2	11/ 4	4/ 1	5 /2	0
**Neurons**	Tip	11/3	7/3	10/3	5/2	24/ 7	10/ 3	10/ 4	13/ 4	5/ 2	11/4
	Porous	21/4	18/4	12/3	10/4	24/ 7	13/ 3	18/ 4	14/ 4	8/ 3	12/4
	Solid	10/3	14/4	4/2	10/4	12/ 7	7 /2	8/ 3	0	4 /2	0

Supplementary Table S1. Immunohistological sample size of analyzed implants and cryosections. Number of examined brain slices (n) / Hemispheres (N). Ten optical sections were made from each 40 μm thick brain slice.

	1 hour	1 day	3 days	1 week	2 weeks	4 weeks	8 weeks
**Microglia Porous-Shells E**	**0.590±0.52**<br>P (1d)-0.27<br>*P (3d)-0.0003<br>*P (1w)-0.0001<br>*P (2w)-8.04E-06<br>*P (4w)-1.35E-08<br>*P (8w)-1.46E-07	**0.50±0.36**<br>P (1h)- 0.27<br>*P (3d)-0.000195<br>*P (1w)-7.02E-05<br>*P (2w)-4.83E-06<br>*P (4w)-1.49E-08<br>*P (8w)-6.64E-09	**2.67±1.28**<br>*P (1h)-0.003<br>*P (1d)-0.000195<br>P (1w)-0.354<br>P (2w)-0.493<br>*P (4w)-0.1468<br>*P (8w)-0.01684	**2.46±1.29**<br>*P (1h)-0.0001<br>*P (1d)-7.02E-05<br>P (3d)-0.354<br>P (2w)-0.332189<br>P (4w)-0.05583<br>*P (8w)-0.023259	**2.67±1.29**<br>*P (1h)-8.04E-06<br>*P (1d)-4.83E-06<br>P (3d)-0.493<br>P (1w)-0.33219<br>P (4w)-0.11542<br>*P (8w)-0.00446	**3.18 ±0.88**<br>*P (1h)-1.35E-08<br>*P (1d)-1.49E-08<br>P (3d)-0.1468<br>P (1w)-0.05583<br>P (2w)- 0.11542<br>*P (8w)-1.2E-05	**1.64±0.52**<br>*P (1h)-1.46E-07<br>*P (1d)-6.64E-09<br>*P (3d)-0.01684<br>*P (1w)-0.02326<br>*P (2w)- 0.00446<br>*P (4w)- 1.2E-05
**Microglia Porous-Shells 1**	**1.17±0.32**<br>P (1d)-0.25<br>*P (3d)-1.584E-07<br>*P (1w)-1.17E-06<br>*P (2w)-2.56E-06<br>*P (4w)-0.000115<br>*P (8w)-0.0058	**1.11±0.32**<br>P (1h)-0.25<br>*P (3d)-1.25E-07<br>*P (1w)-8.13E-07<br>*P (2w)-7.04E-07<br>*P (4w)-4.74E-05<br>*P (8w)-0.000928	**3.93±0.77**<br>*P (1h)- 1.58E-07<br>*P (1d)- 1.25E-07<br>*P (1w)- 0.02898<br>*P (2w)-2.58E-06<br>*P (4w)-1.91E-06<br>*P (8w)-7.94E-07	**3.22±0.93**<br>*P (1h)- 1.17E-06<br>*P (1d)- 8.13E-07<br>*P (3d)- 0.02898<br>*P (2w)-0.000205<br>*P (4w)-0.000166<br>*P (8w)-6.57E-06	**1.97±0.58**<br>*P (1h)- 2.5E-06<br>*P (1d) 7.0E-07-<br>*P (3d)- 2.6E-06<br>*P (1w)- 0.00020<br>P (4w)-0.39349<br>*P (8w)-0.00026	**1.92±0.53**<br>*P (1h)-0.000115<br>*P (1d)-4.74E-05<br>*P (3d)-1.91E-06<br>*P (1w)-0.000166<br>P (2w)- 0.39349<br>*P (8w)-0.00313	**1.42±0.26**<br>*P (1h)-0.0058<br>*P (1d)-0.000928<br>*P (3d)-7.94E-07<br>*P (1w)-6.57E-06<br>*P (2w)- 0.00026<br>*P (4w)- 0.00313
**Astrocytes Porous - Shells E**	**0.26±0.14**<br>P (1d)-0.10<br>*P (3d)-0.0084<br>*P (1w)-0.00399<br>*P (2w)-2.37E-09<br>*P (4w)-1.73E-06<br>*P (8w)-1.58E-10	**0.20±0.14**<br>P (1h)- 0.10<br>*P (3d)-0.004425<br>*P (1w)- 0.00227<br>*P (2w)-9.45E-10<br>*P (4w)-1.37E-06<br>*P (8w)-8.71E-11	**0.64±0.27**<br>*P (1h)-0.0084<br>*P (1d)- 0.004425<br>P (1w)-0.284<br>*P (2w)-2.53E-05<br>*P (4w)-5.88E-06<br>*P (8w)-1.48E-07	**0.74±0.29**<br>*P (1h)- 0.00399<br>*P (1d)- 0.00227<br>P (3d)- 0.284<br>*P (2w)-0.000125<br>*P (4w)-7.14E-06<br>*P (8w)-7.77E-07	**1.56±0.74**<br>*P (1h)- 2.4E-09<br>*P (1d)- 9.4E-10<br>*P (3d)- 2.5E-05<br>*P (1w)- 0.00012<br>*P (4w)-0.00065<br>*P (8w)-0.01825	**2.96±1.20**<br>*P (1h)-1.73E-06<br>*P (1d)-1.37E-06<br>*P (3d)-5.88E-06<br>*P (1w)-7.14E-06<br>*P (2w)- 0.00065<br>*P (8w)-0.00899	**2.01±0.61**<br>*P (1h)- 1.58E-10<br>*P (1d)- 8.71E-11<br>*P (3d)- 1.48E-07<br>*P (1w)- 7.77E-07<br>*P (2w)- 0.01825<br>*P (4w)- 0.00899
**Astrocytes Porous - Shells 1**	**0.68±0.19**<br>*P (1d)-0.038<br>*P (3d)-0.00059<br>*P (1w)-0.000524<br>*P (2w)-7.24E-07<br>*P (4w)-9.8E-07<br>*P (8w)-1.74E-09	**0.58±0.16**<br>*P (1h)-0.038<br>*P (3d)-0.000389<br>*P (1w)-0.000277<br>*P (2w)-1.33E-07<br>*P (4w)-5.06E-06<br>*P (8w)-1.23E-09	**1.31±0.27**<br>*P (1h)-0.00059<br>*P (1d)- 0.000389<br>P (1w)-0.0796<br>*P (2w)-0.0346<br>*P (4w)-7.78E-05<br>*P (8w)-1.27E-06	**1.60±0.36**<br>*P (1h)- 0.000524<br>*P (1d)- 0.000277<br>P (3d)- 0.0796<br>P (2w)-0.355587<br>*P (4w)-0.001279<br>*P (8w)-8.95E-05	**1.68±0.79**<br>*P (1h)- 7.2E-07<br>*P (1d)- 1.3E-07<br>*P (3d)- 0.0346<br>P (1w)- 0.35559<br>*P (4w)-0.00205<br>*P (8w)-8.9E-05	**2.56±0.82**<br>*P (1h)-9.8E-07<br>*P (1d)-5.06E-06<br>*P (3d)-7.78E-05<br>*P (1w)-0.001279<br>*P (2w)- 0.00205<br>P (8w)-0.29014	**2.72±0.82**<br>*P (1h)- 1.74E-09<br>*P (1d)- 1.23E-09<br>*P (3d)- 1.27E-06<br>*P (1w)- 8.95E-05<br>*P (2w)- 8.9E-05<br>P (4w)- 0.29014
**Neurons Porous - Shells E**	**0.36±0.21**<br>*P (1d)-0.0005<br>*P (3d)-0.0059<br>*P (1w)-9.28e-05<br>P (2w)-0.168<br>*P (4w)-0.046<br>P (8w)-0.050063	**0.16±0.14**<br>*P (1h)-0.0005<br>P (3d)-0.294<br>P (1w)-0.344<br>*P (2w)-0.00958<br>*P (4w)-0.01098<br>*P (8w)-0.0123	**0.19±0.15**<br>*P (1h)-0.0059<br>P (1d)-0.294<br>P (1w)-0.179<br>*P (2w)-0.0497<br>*P (4w)-0.081<br>*P (8w)-0.083	**0.14±0.07**<br>*P (1h)- 9.28e-05<br>P (1d)- 0.344<br>P (3d)- 0.179<br>*P (2w)-0.02211<br>*P (4w)-0.00125<br>*P (8w)-0.001478	**0.29±0.22**<br>P (1h)- 0.168<br>*P (1d)- 0.00958<br>*P (3d)- 0.0497<br>*P (1w)- 0.02211<br>P (4w)-0.2851<br>P (8w)-0.29237	**0.26±0.10**<br>*P (1h)-0.046<br>*P (1d)-0.01098<br>P (3d)-0.081<br>*P (1w)- 0.00125<br>P (2w)- 0.2851<br>P (8w)-0.49534	**0.26±0.13**<br>P (1h)- 0.050063<br>*P (1d)- 0.0123<br>P (3d)- 0.083<br>*P (1w) 0.001478<br>P (2w)- 0.29237<br>P (4w)- 0.49534
**Neurons Porous - Shells 1**	**0.74±0.15**<br>*P (1d)-0.026<br>P (3d)-0.112<br>P (1w)-0.228<br>P (2w)-0.214<br>P (4w)-0.144<br>*P (8w)-0.00081	**0.63±0.17**<br>*P (1h)-0.026<br>P (3d)-0.285<br>*P (1w)-0.01541<br>*P (2w)-0.01534<br>*P (4w)- 0.01580<br>*P (8w)-2.3E-05	**0.67±0.15**<br>P (1h)-0.112<br>P (1d)-0.285<br>P (1w)-0.0513<br>P (2w)-0.0506<br>*P (4w)-0.0397<br>*P (8w)-0.000138	**0.78±0.16**<br>P (1h)-0.228<br>*P (1d)-0.01541<br>P (3d)-0.0513<br>P (2w)-0.38603<br>P (4w)-0.323915<br>*P (8w)-0.00937	**0.79±0.28**<br>P (1h)-0.214<br>*P (1d)-0.01534<br>P (3d)-0.0506<br>P (1w)-0.38603<br>P (4w)-0.35861<br>*P (8w)-0.01394	**0.83±0.26**<br>P (1h)- 0.144<br>*P (1d)-0.0158<br>*P (3d)-0.0397<br>P (1w)-0.323915<br>P (2w)- 0.35861<br>P (8w)-0.05254	**0.99±0.26**<br>*P (1h)- 0.00081<br>*P (1d)- 2.3E-05<br>*P (3d)- 0.000138<br>*P (1w)- 0.00937<br>*P (2w)- 0.01394<br>P (4w)-0.05254

Supplementary Table S2. Mean ± one standard derivation of the Normalized Fluorescent Intensity (NFI) for microglia, astrocytes and neuron cell bodies and neurites within the implanted platform and attached to it (referred to as Shell E) and 0-25µm away from the platform's surface (Shell 1) of the perforated segment only. The P values for each t- test comparing the average NFI at different time points after implantation is given as P followed by brackets indicating the time point of comparison (1hour-1h, 1 day- 1d, 3 days- 3d, 1 week- 1w, 2 weeks- 2w, 4 weeks- 4w and 8 weeks- 8w). The t- test was conducted for two- samples assuming unequal variances. $P<0.05$ Indicated by asterisks.
